# Gut Microbiota and Endothelial Dysfunction Markers in Obese Mexican Children and Adolescents

**DOI:** 10.3390/nu10122009

**Published:** 2018-12-19

**Authors:** Khemlal Nirmalkar, Selvasankar Murugesan, María Luisa Pizano-Zárate, Loan Edel Villalobos-Flores, Cristina García-González, Rosa María Morales-Hernández, Jorge Arturo Nuñez-Hernández, Fernando Hernández-Quiroz, María del Socorro Romero-Figueroa, César Hernández-Guerrero, Carlos Hoyo-Vadillo, Jaime García-Mena

**Affiliations:** 1 Departamento de Genética y Biología Molecular, Centro de Investigación y de Estudios Avanzados del Instituto Politécnico Nacional (Cinvestav), Av. Instituto Politécnico Nacional 2508, Ciudad de México 07360, Mexico; nirmalkar@cinvestav.mx (K.N.); selvasankarbio@gmail.com (S.M.); lvillalobos@cinvestav.mx (L.E.V.-F.); fernando.hernandez@cinvestav.mx (F.H.-Q.); 2 Departamento de Farmacología, Centro de Investigación y de Estudios Avanzados del Instituto Politécnico Nacional (Cinvestav), Av. Instituto Politécnico Nacional 2508, Ciudad de México 07360, Mexico; citocromo@cinvestav.mx; 3 Departamento de Nutrición y Bioprogramación, Instituto Nacional de Perinatología, Ciudad de México 11000, Mexico; pizanozarate@yahoo.com (M.L.P.-Z.); n.cristinagarcia@hotmail.com (C.G.-G.); rmh080868@yahoo.com (R.M.M.-H.); 4 Departamento Clínico de Pediatría, Hospital 220 IMSS, Toluca 50150, Mexico; drjanh@hotmail.com; 5 Coordinación de Investigación en Salud, IMSS, Toluca 50000, Mexico; maria.romero@imss.gob.mx; 6 Departamento de Salud, Universidad Iberoamericana, Ciudad de México 01219, Mexico; cesar.hernandez@ibero.mx

**Keywords:** gut microbiota, endothelial dysfunction, obesity, high throughput DNA sequencing, metabolic syndrome, insulin resistance

## Abstract

Obesity is a metabolic disease characterized by low-grade inflammation and accompanied by dyslipidemia and up-regulation of other bioactive molecules, creating a predisposition to endothelial dysfunction and metabolic syndrome. We studied the association between gut microbiota diversity and endothelial dysfunction (EDF) markers in obese Mexican children and adolescents. We examined clinical data including metabolic factors and EDF markers in blood samples. Gut bacterial diversity was characterized by high-throughput sequencing of V3-16S rDNA libraries. Triglycerides, insulin, homeostasis model assessment-insulin resistant (HOMA-IR), leptin, C-reactive protein (CRP), and EDF marker intercellular adhesion molecule 1 (ICAM-1) were significantly higher in obese children and adolescents. Multivariate analysis showed statistically significant positive associations between vascular cell adhesion molecule 1 (VCAM-1) and Veillonellaceae, and between ICAM-1 and *Ruminococcus* in obese children. In obese adolescents, there was a statistically significant positive association between total cholesterol and *Ruminococcus*, and between ICAM-1 and *Bacteroides*. LEfSe analysis showed that the genus *Lactobacillus* and family Coriobacteriaceae were enriched in children, and genera *Collinsella* and *Prevotella* were enriched in obese adolescents. Obese children and adolescents had higher levels of insulin resistance and metabolic syndrome. These results suggest that obese Mexican children and adolescents had increased levels of CRP and a reduction of adiponectin, which causes higher expression of EDF markers, affecting endothelial function and associating with changes in the gut microbiota.

## 1. Introduction

Obesity is a metabolic disorder and a serious global health issue. In 2016, more than 650 million adults were obese, whereas children and adolescents (aged between 5 and 19 years) who were obese exceeded 340 million [1]. In 2015, 32.4% of adults in Mexico were reported as being obese [2], being second only to the United States. In 2016, 15.3% of Mexican children (aged between 5 and 11 years), and 13.9% adolescents (aged between 12 and 19 years) were reported as being obese [3].

The human gut microbiota is associated with obesity [4], and is mainly dominated by two bacterial phyla; Firmicutes and Bacteroidetes, of which Firmicutes is more abundant in obese individuals [4,5]. The gut microbiota harvests energy from dietary fiber through fermentation, producing short-chain fatty acids (SCFAs) such as acetate, propionate, and butyrate, and influencing host energy metabolism [6,7]. The molar ratio of SCFAs acetate, propionate, and butyrate is 60:20:20 in the colon, and this ratio varies from the caecum to the descending colon [8]. Genetically obese ob/ob mice were reported to show higher amounts of SCFAs in the caecum and less in their feces in comparison to their lean littermates [6].

Endothelial dysfunction (EDF) is an impairment of vasodilatation/vasoconstriction, or diminished availability of nitric oxide (NO) [9]. EDF leads to the up-regulation of Reactive Oxygen Species (ROS), C-reactive protein (CRP), and stimulates the secretion of primary proinflammatory cytokines, such as interleukin (IL-1) and tumor necrosis factor-alpha (TNF-α) [9,10]. These cytokines enhance the expression of adhesion molecules such as Intercellular adhesion molecule 1 (ICAM-1), Vascular cell adhesion molecule 1 (VCAM-1), and E-, L-, and P-selectins in the endothelial cells [10], with blood thickening and formation of small plaques. Subsequently, EDF may cause atherosclerosis and other cardiovascular diseases [9]. EDF can be diagnosed by the gold standard method of angiography with acetylcholine injection [11], the Flow Mediated Dilation (non-invasive) method (FMD) [12], or by measurement of EDF markers such as VCAM-1, ICAM-1, and E-selectin [13,14]. In Mexico, around 29.8% of children (aged between 3 and 17 years) have EDF [15]. EDF is also an early marker for atherosclerosis [16]; atherosclerosis was observed in 53% of autopsies in Mexico during 2005–2007 [17].

Obesity was reported to be associated with endothelial dysfunction [18], and atherosclerosis is also associated with endothelial dysfunction [19]. In addition, human gut microbiota is associated with obesity [4] and some specific members of the gut microbiota found in the feces of atherosclerosis patients are also found in their plaques [20,21]. These reports suggest that the gut microbiota may be associated with EDF or EDF markers. Diet is an important factor modulating microbial diversity. It was also reported that high fat diet is associated with obesity, whereas fiber-rich diet can reduce the risk of obesity [22]. As mentioned above, obesity and EDF prevalence are 15.3% and 29.8%, respectively, in Mexican children, and 5–17-year-old obese children have a higher risk of cardiovascular disease [23]. To the best of our knowledge, there is no published report about the association between EDF markers and gut microbiota in any population. Therefore, we aimed to investigate whether there is an association between EDF markers and the intestinal microbiota in obese Mexican children and adolescents [24].

## 2. Materials and Methods

### 2.1. Selection of Study Subjects

A total of 172 individuals was selected, including children (*n* = 111) between the age of 6 and 11 years and adolescents (*n* = 61) between the age of 12 and 18 years. These individuals were divided into two groups: Normal weight (*n* = 49) and obese individuals (*n* = 62) in children, and normal weight (*n* = 27) and obese individuals (*n* = 34) in adolescents. They were selected among children and adolescents attending three different Mexican public schools: Escuela Juan Fernández Albarrán, Centro Escolar Lázaro Cárdenas, Secundaria Técnica Tierra y Libertad, and one hospital (220 IMSS, Instituto Mexicano del Seguro Social), located in the city of Toluca, Mexico. All individuals were interviewed and screened by a certified pediatrician for inclusion and exclusion of participants. All selected participants were healthy with no gastrointestinal diseases or probiotics use in the previous 3 months. The exclusion criteria were: Chronic diseases, smoking, pregnancy, allergies, thyroid disease, eating disorders, consumption of any supplement, atherosclerotic cardiovascular disease, and administration of oral antibiotics in the previous 3 months. Informed consent was obtained from all participants and their parents in accordance with the Helsinki Declaration revised in 2013. The protocol was approved by the Research and Ethical Committee Boards of the Instituto Nacional de Perinatología, 212250-3310-11402-01-14, Mexico City.

### 2.2. Anthropometrical Evaluation

Systolic blood pressure (SBP), diastolic blood pressure (DBP), weight, height, and waist circumference (WC) were measured. Body mass index (BMI) and BMI percentile were calculated and classified based on the World Health Organization (WHO) norms and calculated as weight (kg)/height^2^ (m^2^) [25]. According to this, individuals were classified into two groups: Normal weight (BMI < 85th percentile) and obese individuals (BMI ≥ 95th percentile).

### 2.3. Dietary Profile

Diversity in the diet intake of all participants was assessed using a 7-day dietary recall survey applied by certified dietitians. Diet intake was divided into seven food groups as follows: (1) Starchy staples, (2) legumes, (3) dairy, (4) meat, (5) vitamin A-rich fruits and vegetables, (6) other fruits and vegetables or fruit juices, and (7) foods made with oil, fat, or butter. Food groups that were consumed ≥3 days by each participant in the previous week received a score of 1, and those food groups that were consumed <3 days by each participant in the past week were scored 0. A final score was calculated for each participant adding the values of all the consumed food groups. Thus, a score of 7 was the maximum possible value as previously described [5].

### 2.4. Biochemical Test

Blood samples were collected from each participant after 12 h of fasting in a Vacutainer (BD, Mississauga, Canada) rapid serum tube. Fasting glucose, total cholesterol, high-density lipoprotein (HDL), and triglycerides (enzymatic colorimetric; Diasys, Holzheim, Germany) were analyzed using an automatic analyzer (LORY 2000, Diasys; Diagnostic Systems GmbH, Holzheim, Germany). Friedewald formula was used to calculate low-density lipoprotein (LDL) [26]. C-reactive protein (CRP), insulin, and interleukins were measured by chemiluminescence (Immulite 1000; Siemens Health Care Diagnostic, (Malvern, PA, USA). Leptin and adiponectin concentrations were quantified with the Enzyme-Linked ImmunoSorbent Assay (ELISA), sandwich type (R&D Systems, Minneapolis, MN, USA) [27]. Homeostasis model assessment-insulin resistant (HOMA-IR) was calculated using the formula: Fasting insulin (mU/mL) × fasting glucose (mmol/L)/22.5 for all participants [28,29]. The cut-off value was 2.89 ± 0.7 [28]. A participant was classified as having metabolic syndrome (MetS), if they had at least 3 out of 5 criteria, including WC ≥ 75th percentile; triglycerides ≥ 100 mg/dL; HDL < 50 mg/dL for children, or <45 mg/dL for adolescents; glucose ≥ 100 mg/dL, and blood pressure (SBP/DBP) > 90th percentile for the age and sex categories. These criteria were considered for Mexican children and adolescents based on previous reports [30,31,32,33].

### 2.5. Measurement of EDF Markers

The concentration of EDF markers including sVCAM-1 (soluble vascular cell adhesion molecule, Cat. #DVC00), sICAM-1 (soluble Intercellular adhesion molecule, Cat. #DCD540), and E-selectin (Cat. #DSLE00) were measured in serum of all individuals [14], using quantitative immunoassay technique kit (R&D Systems, Minneapolis, MN, USA).

### 2.6. Collection of Fecal Samples

Fecal samples from both children and adolescents were collected aseptically in a sterile stool container at home in the morning. Once received, samples were immediately transported to the laboratory in cold boxes with ice-gel-packs previously cooled at −70 °C. Samples were aliquoted in multiple tubes and stored at −70 °C.

### 2.7. DNA Extraction and High Throughput Sequencing

DNA was extracted from 100 mg of fecal sample using stool kit method (Favorgen Biotech Corp., Ping-Tung, Taiwan; Favor prep stool kit, Cat. #FASTI001-1). DNA concentration was measured by NanoDrop 2000 spectrophotometer (Thermo Scientific, Waltham, MA, USA) and quality evaluated by 0.5% agarose gel electrophoresis. V3-16S rDNA libraries were prepared by polymerase chain reaction (PCR) and high-throughput sequencing was performed as previously described [5]. Sequences were submitted to National Center for Biotechnology Information (NCBI) BioProject database with accession number PRJNA433269 and can be accessed through the following link: https://www.ncbi.nlm.nih.gov/bioproject/?term=PRJNA433269.

### 2.8. Measurement of SCFAs

SCFAs were measured in 100 mg of dehydrated fecal samples using PerkinElmer-Flexar (Waltham, MA, USA) high performance liquid chromatography (HPLC) equipment as previously reported [5]. Mobile phase consisted of two solutions; 80% of (A) 20mM NaH_2_PO_4_ (Sigma-Aldrich Cat. #S8282, St. Louis, MO, USA) pH 2.2 adjusted with phosphoric acid (J.T. Baker, Estate of Mexico, Xalostoc, Mexico, Cat. #0260-05), and 20% of (B) Acetonitrile (J.T. Baker, Cat. #9012-03, Estate of Mexico, Xalostoc, Mexico), using a 1.0 mL/min flow rate in a 15 cm C-18 column [34]. All chromatographic data were processed using Chromera (v4.1.2.6410, PerkinElmer, Waltham, MA, USA)—HPLC Flexar Software (PerkinElmer, Waltham, MA, USA).

### 2.9. Gut Microbial Diversity

To evaluate the alpha diversity of gut microbial communities, we calculated Shannon, Simpson, and Chao1 indexes and observed species using phyloseq (vegan (v2.2.1), and ggplot2 packages) in the R environment (v3.3.3.). To assess the beta-diversity, dissimilarity index was calculated by UniFrac distance metric, and visualized by principal coordinate analysis as previously described [5].

### 2.10. Gut Microbial Abundance

Linear discriminant analysis (LDA) effect size (LEfSe, v1.0) was used to elucidate significantly different relative abundances of bacterial taxa, associated with both children and adolescents. These analyses are presented in a bar plot and the parameters set with default *p*-value, α = 0.05, and an LDA score of 2.0 with LEfSe [35].

### 2.11. Multivariate Analysis

Multivariate association with linear models (MaAsLin, v0.0.4, was performed to investigate the associations between taxa abundances and clinical metadata using default parameters in *R*. These analyses were used to explore associations between figures reporting *p*- and *q*-values. The false discovery rate (FDR) (*q*-value) was calculated using the Benjamini-Hochberg method to avoid the inclusion of false positives [36]. *p*-values less than 0.05 and *q*-values less than 0.25 were considered significant [37].

### 2.12. Co-Occurrence Analysis

Co-occurrence analysis was performed using *otu_table.biom* files in CoNet (Co-occurrence Network Inference) plugin tool [38], and generated co-occurrence networks were visualized and analyzed in Cytoscape (v3.6.1) software. To avoid false positive results, corrections were made using the Benjamini-Hochberg method (*q*-value). *p*- and *q*-values < 0.05 were considered statistically significant, and correlations analysis (Pearson/Spearman) were sorted for statistically significant (*p* < 0.05) and *R* > 0.8.

### 2.13. Statistical Analysis

The clinical characteristics of all individuals including anthropometric parameters, metabolic factors, EDF markers, SCFAs analysis, and other characteristics were statistically calculated using one-way analysis of variance (ANOVA), and the Mann–Whitney U test. Clinical data are expressed in means ± standard error. Sequencing data were analyzed using Quantitative Insights Into Microbial Ecology (QIIME) pipeline (v1.9.0). Operational taxonomic units (OTUs) picking was run against the Greengenes (v13.8) database. Images were plotted using ggplot2 and RcolorBrewer (v1.1-2) packages. To correct the *p*-values, multiple testing [36] was performed using the *p.adjust* function in *R* to avoid the inclusion of false positives, including alpha-diversity and bacterial relative abundance. Gut bacterial diversity (alpha-diversity) was assessed with phyloseq, and vegan (v2.2-1) packages.

## 3. Results

### 3.1. Obese Children and Adolescents Have Dyslipidemia, Insulin Resistance, and Metabolic Syndrome

We studied a total of 172 individuals divided into two groups by age: 111 children (6–11 years-old) and 61 adolescents (12–18 years-old). Obese children and adolescents weighed significantly more (*p* < 0.001), had higher BMI percentiles (*p* < 0.001), larger WC (*p* < 0.001), and higher WC percentiles (*p* < 0.001) with respect to normal-weight children and adolescents. In addition, SBP (children, *p* = 0.010; adolescents, *p* < 0.001), and DBP (*p* = 0.048) were significantly higher in obese individuals. However, their percentiles were not statistically significant, except the SBP percentile of adolescents (Table 1 and Table 2).

Obese children and adolescents had significantly higher triglycerides (*p* < 0.001) and leptin (*p* < 0.001) levels and lower HDL-cholesterol only in obese children (*p* < 0.001), whereas total cholesterol was higher only in obese adolescents (*p* = 0.041). Obese children and adolescents had significantly higher CRP (children, *p* < 0.001; adolescents, *p* = 0.022) and insulin (*p* < 0.001), but similar fasting glucose levels (children, *p* = 0.223; adolescents, *p* = 0.345). Only obese children had higher TNF-α (*p* = 0.006) but lower levels of adiponectin (*p* = 0.019). The measurement of EDF markers in the participants showed an increase in E-selectin (*p* < 0.001) in both obese groups, and ICAM-1 was significantly increased only in obese adolescents (*p* < 0.001). Only the short-chain fatty acid propionic acid was significantly lower among obese children (*p* = 0.027).

The 7-day recall dietary profile did not show any significant difference between normal weight and obese individuals (Table S1).

Children and adolescents were classified as having MetS if they satisfied three out of five criteria including: WC ≥ 75th percentile, triglycerides ≥ 100 mg/dL, and HDL < 50 mg/dL for children or HDL < 45 mg/dL for adolescents (Table 1 and Table 2). Overall, 15 out of 34 obese children (44.1%) and 30 out of 62 obese adolescents (48.4%) were affected by MetS.

### 3.2. Obese Children and Adolescents Have a Trend in Higher Abundance of Firmicutes and Lower Abundance of Bacteroidetes

To evaluate gut microbial composition, we performed high-throughput DNA sequencing of V3-16S rDNA libraries using fecal DNA from all children and adolescents. We processed 13,095,175 total reads for children and 5,819,206 for adolescents. The average number of reads was 118,389 for normal weight children and 117,647 for obese children; in the case of adolescents, the average number of reads was 102,184 for normal weight and 90,007 for obese subjects.

**Figure 1:**
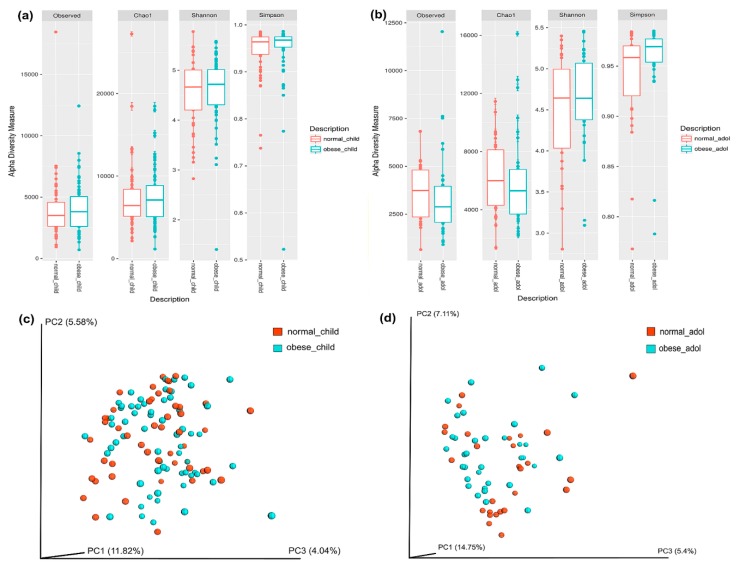
Characterization of alpha-diversity of the gut microbiota in children (**a**), and adolescents (**b**). The y-axes show the Observed numbers of species, Chao1 richness index, and the Shannon and Simpson diversity indexes. The x-axes show the phenotypic categories. Additional data are in Table S2. The gut microbiota beta diversity of normal weight and obese individuals in children (**c**) and adolescents (**d**), was calculated by dissimilarity metrics using operational taxonomic unit (OTU) tables and Unweighted UniFrac analyses. The analysis shows the dissimilarity between normal weight and obese individuals. Three-dimensional scatter plots were generated using principal coordinates analysis (PCoA). Tags beside the graphics are normal weight children, obese children, normal weight adolescents, and obese adolescents. Normal weight individuals are shown in orange, and obese individuals are shown in blue color. Data were analyzed as described in the Materials and Methods section.

The alpha-diversity analysis showed slightly higher diversity in both obese children and adolescents in comparison to normal weight participants; however, differences were not statistically significant (Figure 1 and Table S2). We also calculated the beta-diversity to assess the distance matrix between normal weight and obesity in both children (Figure 1c) and adolescents (Figure 1d). The results of unweighted UniFrac analyses was plotted by principal coordinates analysis (PCoA) and hierarchical clustering. Clustering was not observed.

**Figure 2:**
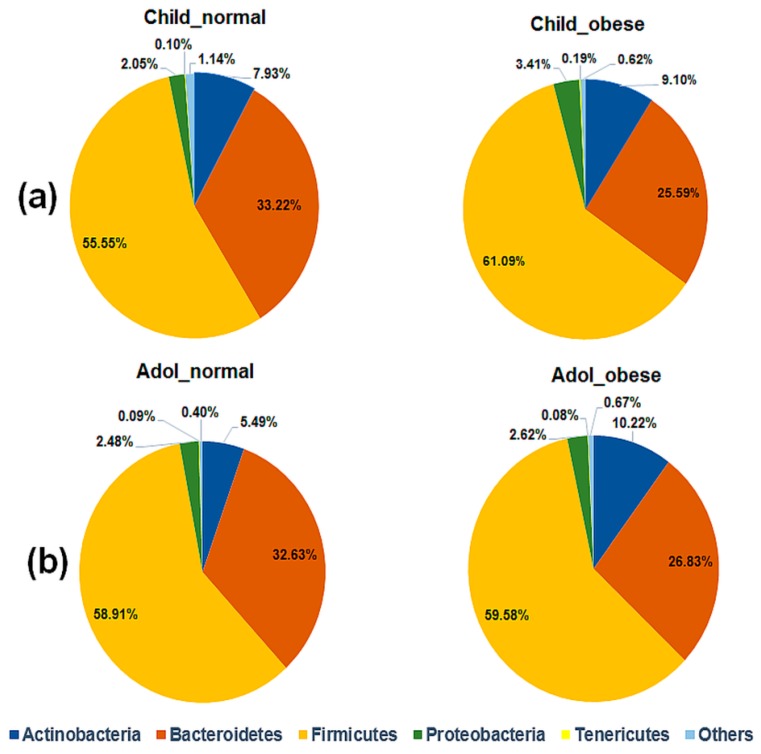
Relative abundance of gut bacterial phyla in children (**a**) and adolescents (**b**). Pie charts shows the gut microbial diversity for each phenotypic group which is indicated on top. The relative abundance of each phylum is shown as percentage (%) beside the charts. Actinobacteria, Bacteroidetes, Firmicutes, Proteobacteria, Tenericutes, and “Others” phyla are indicated by the different colors, described at the bottom of the figure. Tags on top of each chart are normal weight children, obese children, normal weight adolescents and obese adolescents. Additional information is in Table S3.

Next, we evaluated the composition of the gut microbiota at the phylum level. Obese children had higher relative abundance of Firmicutes and Actinobacteria and decreased Bacteroidetes with respect to the normal weight (Figure 2a); however, these differences were not statistically significant (Table S3). Similar results were obtained for Actinobacteria in obese adolescents; however, it was not statistically significant after FDR correction (Figure 2b and Table S3).

### 3.3. Differences in Abundance of Gut Bacteria between Normal Weight and Obese Children and Adolescents

**Figure 3:**
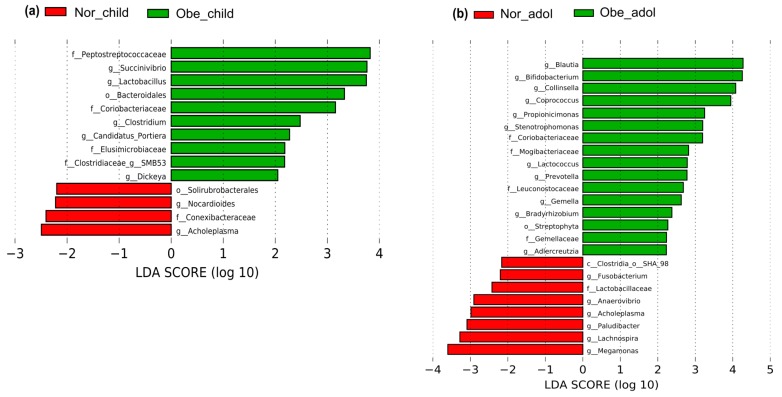
Graphics of Linear discriminant analysis (LDA) effect size (LEfSe) for children (**a**) and adolescents (**b**). Horizontal bars represent the effect size for each taxon. The length of the bar represents the log_10_ transformed LDA score, indicated by vertical dotted lines. Normal weight children and adolescents are indicated by red, and obesity by green. The threshold on the logarithmic LDA score for discriminative features was set to 2.0. The taxon of bacteria with statistically significant change (*p* < 0.05) in the relative abundance is written alongside the horizontal lines. The name of the taxon level is abbreviated as p—phylum; c—class; o—order; f—family, and g—genus. Tags above the graphics are normal weight children, obese children, normal weight adolescents, and obese adolescents. Data were processed as described in Materials and Methods section. Statistically significant values are in additional data Tables S4 and S5.

We next used LEfSe analysis to identify bacteria where the relative abundance was significantly increased or decreased in each phenotypic category. Obese children had members of the phylum Firmicutes, e.g., family Peptostreptococcaceae (*p* = 0.036), and the genus *Lactobacillus* (*p* = 0.040), that were three-fold higher than normal weight children. The genera *Clostridium* (*p* = 0.025) and *SMB53* (*p* = 0.018) were also at least two-fold higher. There was at least a three-fold increase in members of the order Bacteroidales (*p* = 0.019) phylum Bacteroidetes, and members of the family Coriobacteriaceae (*p* = 0.019), phylum Actinobacteria. Members of the phylum Proteobacteria, like the genera *Succinivibrio* (*p* = 0.019), were three-fold higher, whereas the genera Candidatus *Portiera* (*p* = 0.042) and *Dickeya* (*p* = 0.042) were at least two-fold higher. There was at least a two-fold increase in the members of the family Elusimicrobiaceae (*p* = 0.005), phylum Elusimicrobia. In normal weight children, bacteria from the phylum Actinobacteria, order Solirubrobacterales (*p* = 0.010), the family Conexibacteraceae (*p* = 0.022), and the genus *Nocardioides* (*p* = 0.022), and similarly the genus *Acholeplasma* (*p* = 0.049) of the phylum Tenericutes, were at least two-fold more abundant (Figure 3a and Table S4).

Obese adolescents had members of the phylum Firmicutes, e.g., genus *Blautia* (*p* = 0.043) that were at least four-fold higher; in addition, the genus *Coproccous* (*p* = 0.020) was three-fold higher and the families Mogibacteriaceae (*p* = 0.002), Leuconostocaceae (*p* = 0.016), Gemellaceae (*p* = 0.004), as well as the genera *Lactococcus* (*p* < 0.001), and *Gemella* (*p* = 0.005) were at least two-fold higher. Regarding the phylum Bacteroidetes, the abundance of genus *Prevotella* (*p* = 0.030) was at least two-fold higher. Two genera of the phylum Proteobacteria were higher: *Stenotrophomonas* (*p* = 0.013) was three-fold and *Bradyrhizobium* (*p* = 0.049) was at least two-fold higher. Members of the phylum Actinobacteria, like the genera *Bifidobacterium* (*p* = 0.021) and *Collinsella* (*p* = 0.001), were at least four-fold higher; the family Coriobacteriaceae (*p* = 0.003) and the genus *Propiocinimonas* (*p* = 0.039) were also at least three-fold higher, and finally the genus *Adlercreutzia* (*p* = 0.004) increased by at least two-fold. We detected at least a two-fold increase in the phylum Cyanobacteria, order *Streptophyta* (*p* = 0.030). Conversely, in normal weight adolescents, genera from the phylum Firmicutes, like *Lachnospira* (*p* = 0.031) and *Megamonas* (*p* = 0.036), were at least three-fold higher, whereas the order SHA-98 (*p* = 0.024), the family Lactobacillaceae (*p* = 0.002), and the genus *Anaerovibrio* (*p* = 0.048) were at least two-fold higher. There was at least a three-fold increase in genus *Paludibacter* (*p* = 0.040) of the phylum Bacteroidetes, and two additional phyla, Tenericutes and Fusobacteria, had members whose abundances increased at least two-fold: genus *Acholeplasma* (*p* = 0.048) and genus *Fusobacterium* (*p* = 0.032), respectively (Figure 3b and Table S5).

### 3.4. Significant Association between Gut Microbiota Members and EDF and Dyslipidemia Markers in Obesity

**Figure 4:**
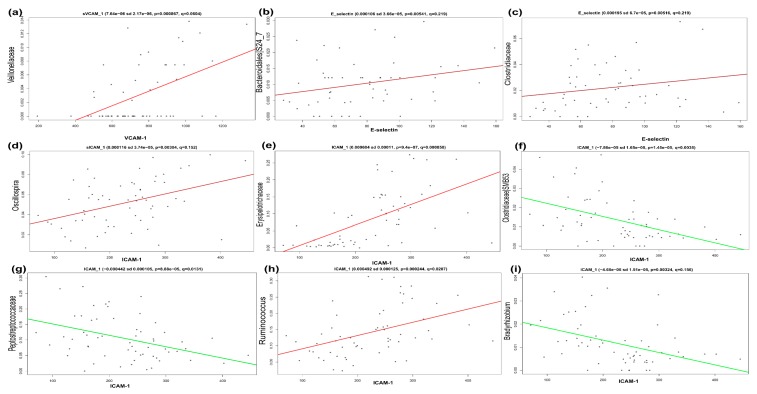
Multivariate linear associations of clinical metadata and bacterial relative abundance in obese children. Scatter plots show the significant associations of vascular cell adhesion molecule 1 (VCAM-1) with Veillonellaceae (**a**), E-selectin with S24-7 (family Bacteroidales) (**b**), E-selectin with Clostridiaceae (**c**), intercellular adhesion molecule 1 (ICAM-1) with *Oscillospira* (**d**), ICAM-1 with Erysipelotrichaceae (**e**), ICAM-1 with SBM53 (**f**), ICAM-1 with Peptostreptococcaceae (**g**), ICAM-1 with *Ruminococcus* (**h**), and ICAM-1 with *Bradyrhizobium* (**i**), as described in Materials and Methods section and Table S6. y-axes show the relative abundance of gut microbiota; x-axes show the clinical metadata. Numerical data on top of each graphic are Coefficient (positive coefficient shows positive association, and negative coefficient shows negative association between metadata and gut microbiota), sd—standard deviation; *p*-values; e—times 10 is raised to the power of, and FDR corrected *q*-values which are assigned by MaAsLin (v0.0.4). VCAM-1-vascular cell adhesion molecule-1, and ICAM-1-intercellular adhesion molecule-1.

**Figure 5:**
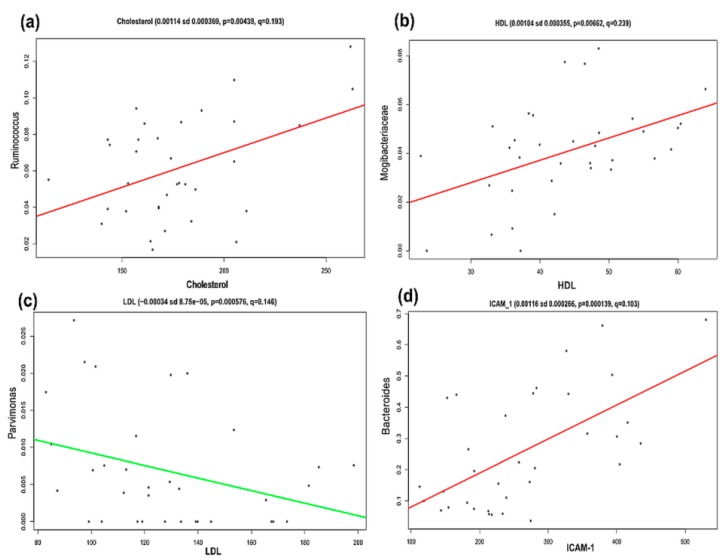
Multivariate linear associations of clinical metadata and bacterial relative abundance in obese adolescents. Scatter plot explains the significant association of Cholesterol with *Ruminococcus* (**a**), HDL with Mogibacteriaceae (**b**), LDL with *Parvimonas* (**c**), and ICAM-1 with *Bacteroides* (**d**), as described in Materials and Methods section and Table S7. *p*-values and FDR corrected *q*-values are assigned by MaAsLin (v0.0.4). y-axes show the relative abundance of gut microbiota; x-axes show the clinical metadata. Numerical data on top of each graphic are Coefficient (positive coefficient shows positive association, and negative coefficient shows negative association between metadata and gut microbiota), sd—standard deviation; e—times 10 is raised to the power of; *p*-values, and FDR corrected *q*-values which are assigned by MaAsLin (v0.0.4). HDL—high-density lipoprotein; LDL—low-density lipoprotein, and ICAM-1—intercellular adhesion molecule-1.

The association between clinical metadata (Table 1 and Table 2) and the relative abundance of gut microbiota was explored via MaAsLin for both children and adolescents. The results showed a positive association in obese children between VCAM-1 and Veillonellaceae (*p* < 0.001, *q* = 0.060), E-selectin and family S24-7 (*p* = 0.005, *q* = 0.219), and between ICAM-1, and *Oscillospira* (*p* = 0.003, *q* = 0.152) (Figure 4). A positive association was also found in obese children between ICAM-1 and *Ruminococcus* (*p* < 0.001, *q* = 0.020). On the other hand, there was a negative association between ICAM-1 and *SMB53* (*p* < 0.001, *q* = 0.003) and between ICAM-1 and Peptostreptococcaceae (*p* < 0.001, *q* = 0.013) in the same individuals. In contrast, obese adolescents had a positive association between total cholesterol and *Ruminococcus* (*p* = 0.004, *q* = 0.193), and between ICAM-1 and *Bacteroides* (*p* = 0.0001, *q* = 0.102). Finally, there was a negative association between LDL and *Parvimonas* (*p* = 0.0012, *q* = 0.146) (Figure 5).

### 3.5. Interactions between Gut Microbiota in Obese Children and Adolescents

**Figure 6:**
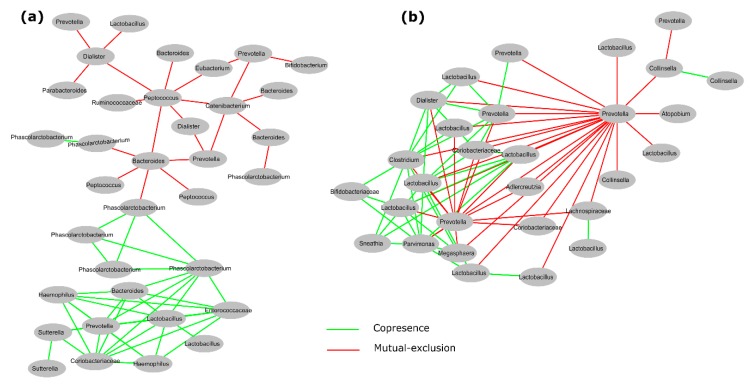
Significant co-occurrence analysis between gut microbiota in obese Mexican children (**a**) and adolescents (**b**). This graphic shows selected interactions between different bacterial communities; copresence (positive, green lines) and mutual exclusion (negative, red lines). This analysis was performed using *otu_table.biom* file in CoNet plugin tool and generated co-occurrence networks were visualized by Cytoscape (v3.6.1) software as described in Material and Methods. Each node indicates a microbial clade (bacterial taxon) belonging to a unique OTUs number. Edges (lines) connecting two nodes, represent significant correlations (*p* < 0.05; *q* < 0.05; *R* > 0.8). In this graphics some bacteria names are shown more than one time e.g., *Lactobacillus, Prevotella*, etc., however they belong to different OTUs number. Bacteria names and corresponding OTU number are shown in Table 3, while a complete set of networks in Figures S3 and S4, and their corresponding OTU number are shown in Table S10, S11, and the significant interaction values are shown in Table S12.

To investigate the interactions between gut microbiota, we performed a co-occurrence analysis as described in Section 2. This analysis showed an interesting network including 35 statistically significant bacterial copresence (positive) and mutual exclusion (negative) interactions (Figure 6a and Table 3) in obese children. Whereas in obese adolescents, it showed 29 statistically significant bacterial copresence (positive) and mutual exclusion (negative) interactions (Figure 6b and Table 3). In contrast, we did not find comparable large complex networks of gut microbiota in normal weight children (Figure S1, Table S8) or adolescents (Figure S2 and Table S9).

## 4. Discussion

Obesity is a metabolic disease characterized by low grade chronic inflammation, usually accompanied by dyslipidemia and up-regulation of other bioactive molecules such as CRP and TNF-α [5,10]. In this work, we studied a sample of Mexican children and adolescents characterizing clinical aspects, EDF markers, and their association with the gut microbial diversity.

Obese children and adolescents had higher BMI percentiles, waist circumference above the 95th percentiles, more hypertriglyceridemia and hypercholesterolemia, and reduced HDL levels (Table 1 and Table 2). In addition, leptin and the percentage of active β-cells were increased in obese children and adolescents. Furthermore, obese children and adolescents presented with greater levels of insulin resistance, as reflected by the elevated glucose, insulin and HOMA-IR values (Table 1 and Table 2). Moreover, these obese children and adolescents had higher blood pressure and metabolic syndrome at this early age.

Among obese children and adolescents there were significant differences for the adipokines, specifically CRP was increased, and adiponectin was decreased among both groups. Surprisingly, TNF-α was only increased among obese children, and not among obese adolescents. For the EDF markers, obese children had significantly elevated levels of E-selectin, though ICAM-1 and VCAM-1 were not significantly increased. In obese adolescents, E-selectin and ICAM-1 were significantly elevated, whereas VCAM-1 was slightly decreased (Table 1 and Table 2). Increased levels of CRP along with reduced levels of adiponectin increased the expression of EDF markers in obese individuals by impairing endothelium-dependent vasodilatation and nitric acid production [10,39]. These adipokines are also associated with insulin resistance, dyslipidemia, atherosclerosis, endothelial dysfunction, and cardiovascular diseases [39,40,41]. We hypothesize that obesity in these children and adolescents increases the risk for the development of these diseases. It has been also reported that hypertriglyceridemia is associated with atherosclerosis and is also predisposition for the development of cardiovascular disease [42].

Gut microbiota and its microbiome are involved in atherosclerosis and obesity in humans [5,21]. The characterization of gut bacterial diversity by high-throughput DNA sequencing of V3-16S rDNA libraries showed higher relative abundance of Firmicutes and lower relative abundance of Bacteroidetes in obese children and adolescents (Figure 2), as has been similarly reported in mice [4], and American [6] and Japanese human guts [43]. However, changes were not statistically significant for our data (Table S3).

To explore the differences in the relative abundance of bacterial taxa, we performed a LEfSe analysis that showed that obese children and adolescents have significant changes in the abundance of various distinct gut bacteria with respect to normal weight (Figure 3 and Tables S4 and S5). For the phylum Actinobacteria, obese children and adolescents showed an increase in the abundance of members of the family Coriobacteriaceae (Table 4). In the feces from mice/hamsters, genera *Eggerthella* and *Enterorhabdus* (family Coriobacteriaceae) were reported to be positively correlated with intrahepatic levels of triglycerides and non-HDL plasma concentrations, suggesting gut barrier and metabolic dysfunction [44] and chronic inflammation [45]. It may be that gut members of the Coriobacteriaceae family are involved with the high levels of triglycerides and cholesterol in obese children. In obese adolescents, the genus *Collinsella* was dominant. This bacterium has been found in plaque and feces of symptomatic atherosclerosis patients [21] and in American rheumatoid arthritis patients [46], which explains that *Collinsella* can be also associated with EDF or EDF markers.

For the phylum Bacteroidetes, members of the order Bacteroidales were more abundant in obese children (Figure 3), similar to what is reported for Sprague-Dawley rats fed a high-fat diet in comparison to rats fed a low-fat diet [47], and in American children and adults affected by inflammatory bowel disease (Table 4) [48]. Additionally, *Prevotella* showed higher abundance in obese adolescents, as observed in American rheumatoid arthritis adult patients. It is suggested that this bacterium can stimulate the epithelial cells to produce IL-8, IL-6, and CCL20, which can promote mucosal Th17 immune responses and neutrophil recruitment and mediate inflammatory reactions [49].

For the phylum Firmicutes, *Lactobacillus* was more abundant in obese children. *Lactobacillus* species were reported to be associated with weight gain in farm animals [50] and French obese adults [51] (Table 4). *Lactobacillus* species are commonly used as probiotics; it is possible that species with increased abundance in obesity are strains with additional genes in the core genome supplied by its pangenome [52]. *Coprococcus* and *Blautia* showed higher abundances only in obese adolescents. An increase in the abundance of these two bacteria has been reported in overweight and obese Mexican children (Table 4) [5].

We were interested in the association of EDF markers (VCAM-1, ICAM-1, and E-selectin) and gut microbiota diversity. For this, we performed a multivariate analysis (MaAsLin), which showed a positive association between some bacteria and clinical data, for example, between the family Veillonellaceae and VCAM-1 (Figure 4a), and between *Ruminococcus* and ICAM-1 (Figure 4h) in obese children (Table S6). For obese adolescents, there was a positive association between *Ruminococcus* and cholesterol (Figure 5a), and between *Bacteroides* and ICAM-1 (Figure 5d and Table S7). In addition, there was a positive association of E-selectin with the S24-7 family of the order Bacteroidales (Figure 4b and Table S6). The order Bacteroidales was increased in obese children in this work (Figure 3a). Adherent Bacteroidales were reported to trigger an inflammatory reaction in individuals with inflammatory bowel disease [48]. Based on the data mentioned above, we propose that these bacteria stimulate the endothelium to produce more EDF markers, which subsequently affect the endothelial function in these studied obese subjects.

We looked for interactions among members of the gut microbiota using co-occurrence analysis. We observed that microbial interaction is different in normal weight children and adolescents, and obese children and adolescents (Figure 6, Figures S1 and S2). For obese children, we found that *Lactobacillus* showed co-presence (positive interaction) with many other bacteria (Figure 6a). LEfSe analysis showed higher abundance of this bacteria in obese children (Figure 3a). It is possible that *Lactobacillus* may have mutualistic relationships, such as syntrophic interactions with other bacteria. Similarly, in obese adolescents, we found that *Prevotella* showed mutual exclusion (negative interaction) with other gut bacteria, especially with *Collinsella* (Figure 6b)*. Prevotella* and *Collinsella* were highly abundant in obese adolescents according to LEfSe analysis (Figure 3b). These negative interactions reflect the trade-off or competition between gut bacteria taxa in the gastrointestinal (GI) tract. We did not find this kind of interaction for normal weight children (Figure S1 and Table S8) or adolescents (Figure S2 and Table S9). Co-occurrence analysis revealed that members of the gut microbiota, especially more abundant bacteria, create large significant networks with other microbiota members in obese children and adolescents. Furthermore, it suggests that these interactions may help a particular group of microbiota to develop ecological dominance and obtain more space and food, and maintain convenient host-microbe interactions inside the gut.

Endothelial dysfunction is an early predisposing factor for atherosclerosis [16], and it has been reported that gut bacteria, including the family Veillonellaceae, genera *Ruminococcus*, and *Bacteroides*, were present in the feces and plaque of adult patients with atherosclerosis [20], and these bacteria were associated with EDF markers in our obese subjects. The genus *Collinsella*, which was highly abundant in obese adolescents in our study (Figure 3 and Table 4 and Table S5), has also been found in feces and plaque of Swedish adult patients with symptomatic atherosclerosis [21], and in American rheumatoid arthritis patients (Table 4) [46]. All this evidence supports that members of the gut microbiota, especially the family Veillonellaceae and genera *Ruminococcus* and *Bacteroides*, are associated with EDF markers in our studied obese subjects and contributing to EDF.

miRNAs have been reported as potential biomarkers for endothelial dysfunction in obese children [53,54]. Vascular microRNA-204 (miR-204) expression is remotely regulated by the microbiome and impairs endothelial function by targeting the Sirtuin1 lysine deacetylase (Sirt1) in mice [55]. Since we observed that some gut microbiota like genera *Ruminococcus*, *Bacteroides*, and family Veillonellaceae were associated with EDF markers in our obese participants, we think that miRNAs may be related to EDF or EDF markers, and their expression would be regulated by gut microbiota in our obese children and adolescents.

To improve endothelial function, dietary fiber, antioxidant-containing food/vegetables [56], or supplementation with inulin or Inulin Like Fructans (ITF) as a prebiotic could be important therapeutic solutions. It has been reported in a mice model that ITF can help improve endothelial function by increasing nitric oxide (NO) synthase and reducing oxidative stress [57]. ITF also improves gut health by increasing NO-producing bacteria and increasing *Akkermansia muciniphila* abundance, which may help reduce the level of EDF markers in patients. In addition, there are many potential pharmacological interventions available, like angiotensin-converting enzyme (ACE)-inhibitors, angiotensin-receptor blocker, calcium channel blockers (CCB), and certain β-blockers, in particular the NO-group (containing molecule nebivolol), which might reverse endothelial dysfunction [56,58]. Limitation on eating high-fat food or consumption of the Western diet is also very important.

With regard to fecal SCFA, its low concentration in obese children and adolescents (Table 1 and Table 2) may be explained by higher mucosal absorption, as has been suggested in other studies [5,6]. We were not able to measure the SCFAs in plasma to confirm the higher absorption due to insufficient blood samples. A strength of our work is that we demonstrated significant changes in gut microbial composition of obese Mexican children and adolescents. Some specific members of the gut microbiota were positively associated with EDF markers in the same individuals affected by obesity. Indeed, this is an emerging field of interest with regards to obesity and pathophysiology and may be helpful for future intervention studies. Our study is not without limitations, including the small sample size, and homogenous cohort. Future studies should include adults, and diverse race/ethnic groups where the prevalence of EDF, atherosclerosis or cardiovascular disease may be higher. Finally, dietary interventions using high-fiber containing foods might be useful for improving endothelial function through the modification of the gut microbiota.

## 5. Conclusions

In conclusion, we find an association between features of the gut microbiota and endothelial function in obese Mexican children and adolescents. Given that an early onset of obesity results in metabolic disorders, targeting the gut microbiota through dietary and therapeutic interventions may be valuable. Furthermore, isolating miRNA's may also provide important information regarding EDF or markers of EDF in obese individuals. Future research targeting improved endothelial function through altered gut microbial health should focus on the role of dietary supplements with high-fiber foods, inulin, or ITF.

## Figures and Tables

**Table 1:** Clinical characteristics of 6–11-year-old children.

Characteristics	Normal Weight	Obesity	*p*-Value
Number (F/M)	49 (30/19)	62 (27/35)	Nd
Age (years)	9.14 ± 0.22	9.50 ± 0.18	0.146 ^µ^
Age Range (years)	7–11	6–11	Nd
**Anthropometric**			
Weight (kg)	31.49 ± 1.02	48.54 ± 1.37	<0.001 ^µ^
Height (m)	1.35 ± 0.01	1.39 ± 0.01	0.094 ^Ω^
BMI (kg/m^2^)	16.89 ± 0.21	24.90 ± 0.42	<0.001 ^µ^
BMI pc	57.44 ± 2.71	98.78 ± 0.18	<0.001 ^µ^
BMI pc Scale	<85	>95	Nd
WC (cm)	59.53 ± 0.68	80.55 ± 1.29	<0.001 ^µ^
WC pc	59.46 ± 3.78	97.47 ± 0.72	<0.001 ^µ^
**Blood Pressure**			
SBP (mm Hg)	88.42 ± 3.64	100.61 ± 1.78	0.010 ^µ^
SBP pc	33.20 ± 3.69	38.96 ± 3.53	0.312 ^µ^
DBP (mm Hg)	59.44 ± 2.66	65.56 ± 1.42	0.048 ^µ^
DBP pc	56.56 ± 3.87	64.54 ± 2.86	0.123 ^µ^
**Metabolic Factors**			
Fasting Glucose (mg/dL)	89.04 ± 0.99	91.32 ± 1.33	0.223 ^µ^
Triglycerides (mg/dL)	84.64 ± 5.30	119.73 ± 7.74	<0.001 ^µ^
Total Cholesterol (mg/dL)	162.88 ± 3.45	166.62 ± 2.70	0.651 ^µ^
HDL (mg/dL)	53.25 ± 1.57	46.34 ± 2.16	<0.001 ^µ^
LDL (mg/dL)	92.70 ± 2.82	96.34 ± 2.67	0.394 ^µ^
Insulin (µIU/mL)	6.49 ± 0.61 ^∞^	13.53 ± 1.42	<0.001 ^µ^
HOMA-IR	1.44 ± 0.14	3.12 ± 0.33	<0.001 ^µ^
HOMA-β (%)	91.89 ± 8.86	148.49 ± 34.61	<0.001 ^µ^
**Adipokines & Inflammatory Markers**			
CRP (mg/L)	1.03 ± 0.21 *	4.05 ± 0.87 **	<0.001 ^µ^
IL-1β (pg/mL)	2.40 ± 0.10 ^α^	2.58 ± 1.55 ^αα^	0.879 ^Ω^
IL-6 (pg/mL)	2.83 ± 0.36 ^β^	3.03 ± 0.23 ^ββ^	0.192 ^Ω^
TNF-α (pg/mL)	12.45 ± 0.73 ^€^	16.32 ± 1.70 ^€€^	0.006 ^Ω^
Adiponectin (µg/mL)	13.86 ± 0.81 ^£^	11.24 ± 0.61	0.019 ^Ω^
Leptin (ng/mL)	6.71 ± 0.69 ^¥^	25.11 ± 1.98	<0.001 ^µ^
**Endothelial Dysfunction Markers**			
VCAM-1 (ng/mL)	761.73 ± 31.60	789.37 ± 34.93	0.771 ^Ω^
ICAM-1 (ng/mL)	205.36 ± 8.62	246.81 ± 19.16	0.137 ^Ω^
E-selectin (ng/mL)	58.09 ± 4.53	175.88 ± 71.52	<0.001 ^Ω^
**Fecal SCFAs**			
Acetic Acid (mM/100 mg)	237.84 ± 20.19	221.80 ± 17.14	0.489 ^Ω^
Propionic Acid (mM/100 mg)	14.57 ± 1.91	11.68 ± 2.07	0.027 ^Ω^
Butyric Acid (mM/100 mg)	13.81 ± 1.44	12.18 ± 1.08	0.289 ^Ω^

The results are presented as mean ± standard error. *p*-values were calculated according to: ^µ^ Mann–Whitney U test for unequal variances, ^Ω^ One-way ANOVA for equal variance. *p* < 0.05 are considered statistically significant. Abbreviations are F—female, M—male, BMI—body mass index, WC—waist circumference, SBP—systolic blood pressure, DBP—diastolic blood pressure, HDL—high-density lipoprotein, LDL—low-density lipoprotein, HOMA-IR—homeostasis model assessment-insulin resistant, , HOMA-β—homeostasis model assessment-beta cell function, CRP—C-reactive protein, IL —interleukin, TNF—tumor necrosis factor, VCAM—vascular adhesion molecule, ICAM—intercellular adhesion molecule, SCFAs—short chain fatty acids, pc—percentile, Nd—not determined. Different symbols show the number of participants for the data: ^∞^ 48, ^¥^ 48, * 35, ^α^ 25, ^β^ 25, ^€^ 25, ^£^ 48, out of 49 for Normal weight; or ** 56, ^αα^ 41, ^ββ^ 41, ^€€^ 42, out of 62 for Obesity. WC pc was adjusted according to sex and age.

**Table 2:** Clinical characteristics of 12–18-year-old adolescents.

Characteristics	Normal Weight	Obesity	*p*-Value
Number (F/M)	27 (12/15)	34 (18/16)	Nd
Age (years)	13.00 ± 0.28	13.61 ± 0.28	0.254 ^µ^
Age Range (years)	12–16	12–18	Nd
**Anthropometric**			
Weight (kg)	44.83 ± 1.54	69.43 ± 2.46	<0.001 ^Ω^
Height (m)	1.52 ± 0.01	1.55 ± 0.01	0.224 ^Ω^
BMI (kg/m^2^)	19.25 ± 0.42	28.78 ± 0.83	<0.001 ^Ω^
BMI pc	52.60 ± 4.34	98.01 ± 0.36	<0.001 ^µ^
BMI pc Scale	<85	>95	Nd
WC	68.91 ± 1.27	88.68 ± 1.57	<0.001 ^µ^
WC pc	65.59 ± 5.53	97.93 ± 0.76	<0.001 ^µ^
**Blood Pressure**			
SBP (mm Hg)	93.84 ± 2.22	104.48 ± 1.73	<0.001 ^µ^
SBP pc	20.49 ± 3.90	36.51 ± 4.73	0.021 ^µ^
DBP (mm Hg)	63.12 ± 1.41	68.30 ± 1.77	0.048 ^µ^
DBP pc	51.20 ± 3.89	62.52 ± 4.50	0.074 ^µ^
**Metabolic Factors**			
Fasting Glucose (mg/dL)	91.45 ± 2.07	92.89 ± 1.68	0.345 ^µ^
Triglycerides (mg/dL)	91.14 ± 5.67	136.94 ± 8.33	<0.001 ^µ^
Total Cholesterol (mg/dL)	161.98 ± 2.64	176.93 ± 5.20	0.041 ^µ^
HDL (mg/dL)	48.61 ± 1.84	43.86 ± 1.70	0.069 ^Ω^
LDL (mg/dL)	95.13 ± 2.35	105.68 ± 4.30	0.089 ^µ^
Insulin (µIU/mL)	8.64 ± 0.67	17.69 ± 1.51	<0.001 ^µ^
HOMA-IR	1.93 ± 0.14	4.07 ± 0.36	<0.001 ^µ^
HOMA-β (%)	126.69 ± 14.67	256.49 ± 38.70	<0.001 ^µ^
**Adipokines & Inflammatory Markers**			
CRP (mg/L)	2.27 ± 0.76 *	3.15 ± 0.80	0.022 ^µ^
IL-1β (pg/mL)	2.40 ± 0.20 ^∞^	2.83 ± 0.44 ^∞∞^	0.926 ^µ^
IL-6 (pg/mL)	2.74 ± 0.25 ^α^	3.26 ± 0.51 ^αα^	0.824 ^µ^
TNF-α (pg/mL)	13.50 ± 1.23 ^β^	11.12 ± 0.86 ^ββ^	0.092 ^µ^
Adiponectin (µg/mL)	12.50 ± 1.08	10.49 ± 0.86	0.105 ^µ^
Leptin (ng/mL)	14.73 ± 4.19	34.13 ± 4.61	<0.001 ^µ^
**Endothelial Dysfunction Markers**			
VCAM-1 (ng/mL)	799.69 ± 47.67	792.23 ± 60.90	0.127 ^µ^
ICAM-1 (ng/mL)	182.10 ± 17.05	263.11 ± 17.40	<0.001 ^µ^
E-selectin (ng/mL)	44.33 ± 3.35	103.12 ± 19.53	<0.001 ^µ^
**Fecal SCFAs**			
Acetic Acid (mM/100mg)	245.10 ± 28.42	238.76 ± 17.76	0.576 ^µ^
Propionic Acid (mM/100mg)	14.79 ± 3.25	8.50 ± 1.56	0.379 ^µ^
Butyric Acid (mM/100mg)	13.60 ± 1.82	16.65 ± 3.71	0.596 ^µ^

The results are presented as mean ± standard error. *p*-values were calculated according to: ^µ^ Mann—Whitney U test for unequal variances, ^Ω^ One-way ANOVA for equal variance. *p* < 0.05 are considered statistically significant. Abbreviations are F—female, M—male, BMI—body mass index, WC—waist circumference, SBP—systolic blood pressure, DBP—diastolic blood pressure, HDL—high-density lipoprotein, LDL—low-density lipoprotein, HOMA-IR—homeostasis model assessment-insulin resistant, HOMA-β—homeostasis model assessment-beta cell function, CRP—C-reactive protein, IL—interleukin, TNF—tumor necrosis factor, VCAM—vascular adhesion molecule, ICAM—intercellular adhesion molecule, SCFAs—short chain fatty acids, pc—percentile, Nd—not determined. In some studies, not all individuals could participate due to unavailability of samples. Different symbols show the number of participants for the data: * 26, ^∞^ 22, ^α^ 22, ^β^ 22, out of 27 for Normal weight; or ^∞∞^ 30, ^αα^ 30, ^ββ^ 30 out of 34 for Obesity. WC pc was adjusted according to sex and age.

**Table 3:** List of bacterial taxa with their operational taxonomic unit (OTU) IDs for Figure 6.

	Obese Children (Figure 6a)			Obese Adolescents (Figure 6b)	
No.	OTUs ID	Bacteria	No.	OTUs ID	Bacteria
1	335	*Phascolarctobacterium*	1	1184	*Collinsella*
2	4007	*Peptococcus*	2	7367	*Prevotella*
3	6529	*Lactobacillus*	3	9022	*Lactobacillus*
4	7389	*Haemophilus*	4	9328	*Collinsella*
5	9453	*Prevotella*	5	10278	*Lactobacillus*
6	11321	*Phascolarctobacterium*	6	10560	*Collinsella*
7	12345	*Phascolarctobacterium*	7	12479	*Lactobacillus*
8	13188	*Prevotella*	8	12723	Lachnospiraceae
9	19296	*Bacteroides*	9	12974	*Lactobacillus*
10	19314	*Parabacteroides*	10	29566	*Sneathia*
11	21736	*Phascolarctobacterium*	11	128300	*Lactobacillus*
12	22231	*Haemophilus*	12	130468	*Lactobacillus*
13	24722	*Bacteroides*	13	130864	*Lactobacillus*
14	41229	*Sutterella*	14	133372	*Parvimonas*
15	157424	*Phascolarctobacterium*	15	137183	Bifidobacteriaceae
16	179261	*Sutterella*	16	225846	*Dialister*
17	183603	*Bacteroides*	17	236308	*Lactobacillus*
18	196604	*Catenibacterium*	18	272516	*Adlercreutzia*
19	215331	*Peptococcus*	19	292921	*Prevotella*
20	235591	*Lactobacillus*	20	354905	*Lactobacillus*
21	269937	*Prevotella*	21	383885	*Lactobacillus*
22	293883	*Phascolarctobacterium*	22	469663	*Atopobium*
23	309133	Enterococcaceae	23	566154	Coriobacteriaceae
24	339685	*Peptococcus*	24	568118	*Prevotella*
25	365496	*Bacteroides*	25	663885	*Prevotella*
26	370086	Ruminococcaceae	26	840914	*Prevotella*
27	403701	*Dialister*	27	851726	*Megasphaera*
28	524371	*Prevotella*	28	858535	Coriobacteriaceae
29	524884	*Eubacterium*	29	986513	*Clostridium*
30	583746	*Dialister*			
31	587753	Coriobacteriaceae			
32	639310	*Bifidobacterium*			
33	716286	*Lactobacillus*			
34	850218	*Phascolarctobacterium*			
35	4226929	*Bacteroides*			

**Table 4:** Selected gut bacteria with significant changes in abundance according to linear discriminant analysis (LDA) effect size (LEfSe) analysis.

Taxa	This Work	Other Reports	Reference
**Phylum Actinobacteria**			
Family Coriobacteriaceae	3-fold more abundant in obese children and adolescents than normal weight	Higher abundance in human and mouse gut. Involved in bile acid metabolism and linked to gut barrier and metabolic dysfunctions	[44]
		Isolated from the gut of Crohn’s disease suffering adult patient from Germany	[45]
*Collinsella*	4-fold more abundant in obese adolescents than normal weight	More than 3-fold enriched in Swedish adult patients with symptomatic atherosclerosis	[21]
		Abundant in American rheumatoid arthritis patients, strongly correlated with production of pro-inflammatory molecules and alters the gut permeability	[46]
**Phylum Bacteroidetes**			
Order Bacteroidales	3-fold more abundant in obese children than normal weight	Higher abundance in high-fat diet (HFD) fed Sprague-Dawley rats compared with low fat diet (LFD) fed rats (LFD vs. HFD, *p* < 0.01)	[47]
		Reported in intestinal biopsies of American children and adults with Inflammatory Bowel Disease	[48]
*Prevotella*	2-fold more abundant in obese adolescents than normal weight	Higher abundance of *Prevotella* in American adult rheumatoid arthritis patients. Induces inflammatory reactions by stimulating epithelial cells and production of interleukins	[49]
**Phylum Firmicutes**			
*Lactobacillus*	3-fold more abundant in obese children than normal weight	Different *Lactobacillus* species are associated with weight gain in farm animals	[50]
		Higher abundance in obese French adults than normal weight	[51]
*Blautia*	4-fold more abundant in obese adolescents than normal weight	Found higher abundance of *Blautia* in overweight and obese Mexican children	[5]
*Coprococcus*	3-fold more abundant in obese adolescents than normal weight	Reported higher abundance of *Coprococcus* in overweight and obese Mexican children	[5]

## Supplementary Materials

The following are available online at http://www.mdpi.com/2072-6643/10/12/2009/s1, Table S1: Dietary diversity of the studied children and adolescents by phenotypic classification, Table S2: Diversity indexes for children and adolescents, Table S3: Significant level of bacterial phylum in children, Table S4: Linear discriminant analysis (LDA) effect size (LEfSe) analysis for children, Table S5: Linear discriminant analysis (LDA) effect size (LEfSe) analysis for adolescents, Table S6: Taxonomic composition of gut microbiota with different metadata in obese children, Table S7: Taxonomic composition of gut microbiota with different metadata in obese adolescents, Table S8: List of bacterial taxa with their OTUs ID in normal weight children for Figure S1, Table S9: List of bacterial taxa with their OTUs ID in normal weight adolescents for Figure S2, Table S10: List of bacterial taxa with their OTUs ID in obese children for Figure S3, Table S11: List of bacterial taxa with their OTUs ID in obese adolescents for Figure S4, Table S12: Significant values for Co-occurrence analysis. Figure S1: Significant co-occurrence analysis between gut microbiota in normal weight Mexican children, Figure S2: Significant co-occurrence analysis between gut microbiota in normal weight Mexican adolescents, Figure S3: Significant co-occurrence analysis between gut microbiota in obese Mexican children, and Figure S4: Significant co-occurrence analysis between gut microbiota in obese Mexican adolescents.
