# Supplementary material for: Gut Microbiota and Endothelial Dysfunction Markers in Obese Mexican Children and Adolescents
Source: Nutrients. 2018 Dec 19;10(12):2009. doi: 10.3390/nu10122009 (PMC6315777; doi:10.3390/nu10122009)
Supplement: Supplementary file 1 [file nutrients-10-02009-s001.zip › supplementary/Supplementary material-1.pdf]

Article

Gut Microbiota and Endothelial Dysfunction Markers in Obese Mexican Children and Adolescents

Khemlal Nirmalkar[1,2], Selvasankar Murugesan[1,#], María Luisa Pizano-Zárate[3], Loan Edel Villalobos-Flores[1], Cristina García González[3], Rosa María Morales-Hernández[3], Jorge Arturo Nuñez-Hernández[4], Fernando Hernández-Quiroz[1], María del Socorro Romero-Figueroa[5], César Hernández-Guerrero[6], Carlos Hoyo-Vadillo[2], Jaime García-Mena[1*]

[1] Departamento de Genética y Biología Molecular, Centro de Investigación y de Estudios Avanzados (Cinvestav), Av. Instituto Politécnico Nacional 2508, Ciudad de México, Mexico; nirmalkar@cinvestav.mx (K.N.); selvasankarbio@gmail.com (S.M.); lvillalobos@cinvestav.mx (L.E.V.F.); fernando.hernandez@cinvestav.mx (F.H.Q.); jgmena@cinvestav.mx (J.G.M.).

[2] Departamento de Farmacología, Centro de Investigación y de Estudios Avanzados (Cinvestav), Av. Instituto Politécnico Nacional 2508, Ciudad de México, Mexico; citocromo@cinvestav.mx (C.H.V.).

[3] Departamento de Nutrición y Bioprogramación. Instituto Nacional de Perinatología, Ciudad de México, Mexico; pizanozarate@yahoo.com (M.L.P.Z.); n.cristinagarcia@hotmail.com (C.G.G.); rmh080868@yahoo.com (R.M.M.H.).

[4] Departamento Clínico de Pediatría, Hospital 220 IMSS, Toluca, Mexico; drjanh@hotmail.com (J.A.N.H.).

[5] Coordinación de Investigación en Salud, IMSS, Toluca, Mexico; maria.romero@imss.gob.mx (M.S.R.F.).

[6] Departamento de Salud, Universidad Iberoamericana, Ciudad de México, Mexico; cesar.hernandez@ibero.mx (C.H.G.).

* Correspondence: jgmena@cinvestav.mx (J.G.M.); Tel.: +52-5747-3800 (ext. 5328)

\# Present address: Division of Translational Medicine, Research Department, Sidra Medical and Research Institute, Doha, Qatar

Supplementary material

Table S1. Dietary diversity of the studied children and adolescents by phenotypic classification.

Group	Mean diversity score (range 0–7)	% with low diversity	% with middle diversity	% with high diversity
		0–2 food groups	3–4 food groups	5–7 food groups
Children (105)				
Normal weight (46)	6.87	26.71	31.68	41.61
Obesity (59)	6.75	21.18	32.45	42.37
Adolescents (57)				
Normal weight (25)	6.88	26.29	34.86	38.86
Obesity (32)	6.53	32.59	25.00	42.41

Food groups: (1) starchy staples; (2) legumes; (3) dairy; (4) meat; (5) vitamin A-rich fruits and vegetables; (6) other fruits and vegetables or fruit juices; and (7) foods made with oil, fat, or butter. 6 children and 4 adolescents could not participate for dietary diversity study.

Table S2. Diversity indexes for children and adolescents.

Diversity index				
Children	Normal weight*	Obesity*	*p*-value	*q*-value
Observed	4,015.55	4,007.79	0.6995	1.0
Chao1	7,424.22	7,636.18	0.4472	1.0
Shannon	4.53	4.61	0.4946	1.0
Simpson	0.94	0.95	0.4615	1.0
Adolescents	Normal weight*	Obesity*	*p* value	*q* value
Observed	3,569.93	3,412.79	0.3876	1.0
Chao1	6,209.36	5,912.67	0.3876	1.0
Shannon	4.48	4.62	0.4904	1.0
Simpson	0.94	0.95	0.0827	1.0

p-values were calculated by Mann-Whitney test for unequal variances. *p*-values corrected by Benjamini-Hochberg, 1995 method and generated FDR value (*q*-value). $p < 0.05$ and $q < 0.05$ are considered statistically significant. Where * shows mean values

Table S3. Significant level of bacterial phylum in children.

Taxa	Normal weight*	Obesity*	*P*-value	*q*-value
Children				
p_Actinobacteria	7.93%	9.10%	0.1314	0.6906
p_Bacteroidetes	33.22%	25.59%	0.0493	0.6906
p_Firmicutes	55.55%	61.09%	0.0543	0.6906
p_Proteobacteria	2.05%	3.41%	0.2444	0.6906
p_Tenericutes	0.10%	0.19%	0.5328	0.7991
Others	1.00%	0.67%	0.7170	1.0000
Adolescents				
p_Actinobacteria	4.34%	10.04%	0.0025	0.0740
p_Bacteroidetes	32.63%	26.83%	0.3270	0.9771
p_Firmicutes	58.91%	59.58%	0.9248	0.9850
p_Proteobacteria	2.13%	2.06%	0.4723	0.9836
p_Tenericutes	0.09%	0.04%	0.6454	0.9836
Others	0.40%	0.67%	0.7930	1.0000

p-values corrected by Benjamini-Hochberg, 1995 method and generated FDR value (q-value). $p < 0.05$ and $q < 0.05$ are considered statistically significant. The name of a higher taxon level was added before its taxon abbreviation "p", phylum. * shows mean values.

Table S4. Linear discriminant analysis (LDA) effect size (LEfSe) analysis for children.

Taxa	Group	LDA score	*p*-value
p_Firmicutes;c_Bacilli;o_Lactobacillales;f_Lactobacillaceae;g_*Lactobacillus*	Obese children	3.7495	0.0402
p_Proteobacteria;c_Gammaproteobacteria;o_Aeromonadales;f_Succinivibrionaceae;g_*Succinivibrio*	Obese children	3.7613	0.0194
p_Firmicutes;c_Clostridia;o_Clostridiales;f_Clostridiaceae;g_*Clostridium*	Obese children	2.4784	0.0256
p_Elusimicrobia;c_Elusimicrobia;o_Elusimicrobiales;f_Elusimicrobiaceae	Obese children	2.1828	0.0053
p_Actinobacteria;c_Coriobacteriia;o_Coriobacteriales;f_Coriobacteriaceae	Obese children	3.1561	0.0194
p_Firmicutes;c_Clostridia;o_Clostridiales;f_Peptostreptococcaceae	Obese children	3.8233	0.0363
p_Firmicutes;c_Clostridia;o_Clostridiales;f_Clostridiaceae;g_*SMB53*	Obese children	2.1799	0.0180
p_Proteobacteria;c_Gammaproteobacteria;o_Enterobacteriales;f_Enterobacteriaceae;g_*Dickeya*	Obese children	2.0485	0.0429
p_Bacteroidetes;c_Bacteroidia;o_Bacteroidales	Obese children	3.3284	0.0196
p_Proteobacteria;c_Gammaproteobacteria;o_Oceanospirillales;f_Halomonadaceae;g_Candidatus *portiera*	Obese children	2.2739	0.0429
p_Tenericutes;c_Mollicutes;o_Acholeplasmatales;f_Acholeplasmataceae;g_*Acholeplasma*	Normal weight children	2.4934	0.0493
p_Actinobacteria;c_Actinobacteria;o_Actinomycetales;f_Nocardioidaceae;g_*Nocardioides*	Normal weight children	2.2210	0.0226
p_Actinobacteria;c_Thermoleophilia;o_Solirubrobacterales	Normal weight children	2.1983	0.0104
p_Actinobacteria;c_Thermoleophilia;o_Solirubrobacterales;f_Conexibacteraceae	Normal weight children	2.4045	0.0226

The threshold on the logarithmic LDA score for discriminative features was set to 2.0. The name of a higher taxon level was added before its taxon abbreviation. "p", phylum; "c", class; "o", order; "f", family; "g", genus. "LDA" Linear discriminant analysis. $p < 0.05$ are considered statistically significant.

Table S5. Linear discriminant analysis (LDA) effect size (LEfSe) analysis for adolescents.

Taxa	Group	LDA score	*p*-value
p_Actinobacteria;c_Coriobacteriia;o_Coriobacteriales;f_Coriobacteriaceae;g_*Adlercreutzia*	Obese adolescents	2.2280	0.0041
p_Proteobacteria;c_Alphaproteobacteria;o_Rhizobiales;f_Bradyrhizobiaceae;g_*Bradyrhizobium*	Obese adolescents	2.3751	0.0499
p_Actinobacteria;c_Actinobacteria;o_Bifidobacteriales;f_Bifidobacteriaceae;g_*Bifidobacterium*	Obese adolescents	4.2532	0.0210
p_Firmicutes;c_Bacilli;o_Lactobacillales;f_Leuconostocaceae	Obese adolescents	2.6803	0.0160
p_Firmicutes;c_Clostridia;o_Clostridiales;f_Lachnospiraceae;g_*Blautia*	Obese adolescents	4.2739	0.0436
p_Actinobacteria;c_Coriobacteriia;o_Coriobacteriales;f_Coriobacteriaceae;g_*Collinsella*	Obese adolescents	4.0767	0.0018
p_Actinobacteria;c_Coriobacteriia;o_Coriobacteriales;f_Coriobacteriaceae	Obese adolescents	3.1937	0.0035
p_Firmicutes;c_Bacilli;o_Lactobacillales;f_Streptococcaceae;g_*Lactococcus*	Obese adolescents	2.7848	<0.001
p_Firmicutes;c_Clostridia;o_Clostridiales;f_Lachnospiraceae;g_*Coprococcus*	Obese adolescents	3.9461	0.0202
p_Cyanobacteria;c_Chloroplast;o_Streptophyta	Obese adolescents	2.2671	0.0303
p_Bacteroidetes;c_Bacteroidia;o_Bacteroidales;f_Paraprevotellaceae;g_*Prevotella*	Obese adolescents	2.7781	0.0309
p_Firmicutes;c_Clostridia;o_Clostridiales;f_Mogibacteriaceae	Obese adolescents	2.8203	0.0024
p_Firmicutes;c_Bacilli;o_Gemellales;f_Gemellaceae	Obese adolescents	2.2309	0.0040
p_Actinobacteria;c_Actinobacteria;o_Actinomycetales;f_Nocardioidaceae;g_*Propionicimonas*	Obese adolescents	3.2475	0.0394
p_Firmicutes;c_Bacilli;o_Gemellales;f_Gemellaceae;g_*Gemella*	Obese adolescents	2.6242	0.0058
p_Proteobacteria;c_Gammaproteobacteria;o_Xanthomonadales;f_Xanthomonadaceae;g_*Stenotrophomonas*	Obese adolescents	3.1971	0.0132
p_Bacteroidetes;c_Bacteroidia;o_Bacteroidales;f_Porphyromonadaceae;g_*Paludibacter*	Normal weight adolescents	3.0903	0.0403
p_Tenericutes;c_Mollicutes;o_Acholeplasmatales;f_Acholeplasmataceae;g_*Acholeplasma*	Normal weight adolescents	2.9836	0.0481
p_Firmicutes;c_Clostridia;o_Clostridiales;f_Veillonellaceae;g_*Megamonas*	Normal weight adolescents	3.6010	0.0361
p_Firmicutes;c_Clostridia;o_Clostridiales;f_Lachnospiraceae;g_*Lachnospira*	Normal weight adolescents	3.2791	0.0316
p_Firmicutes;c_Clostridia;o_Clostridiales;f_Veillonellaceae;g_*Anaerovibrio*	Normal weight adolescents	2.9063	0.0481
p_Firmicutes;c_Clostridia;o_SHA_98	Normal weight adolescents	2.1641	0.0249
p_Fusobacteria;c_Fusobacteriia;o_Fusobacteriales;f_Fusobacteriaceae;g_*Fusobacterium*	Normal weight adolescents	2.2006	0.0321
p_Firmicutes;c_Bacilli;o_Lactobacillales;f_Lactobacillaceae	Normal weight adolescents	2.4231	0.0024

The threshold on the logarithmic LDA score for discriminative features was set to 2.0. The name of a higher taxon level was added before its taxon abbreviation. “p”, phylum; “c”, class; “o”, order; “f”, family; “g”, genus. “LDA” Linear discriminant analysis. $p < 0.05$ are considered statistically significant.

Table S6. Taxonomic composition of gut microbiota with different metadata in obese children.

Variable	Feature	Coefficient	*p*-value	*q*-value
VCAM-1	p_Firmicutes;c_Clostridia;o_Clostridiales;f_Veillonellaceae	<-0.00010	<0.001	0.060
E-selectin	p_Firmicutes;c_Clostridia;o_Clostridiales;f_Clostridiaceae	0.00019	0.005	0.219
E-selectin	o_Bacteroidales;f_S24-7	0.00010	0.005	0.219
ICAM-1	p_Firmicutes;c_Clostridia;o_Clostridiales;f_Ruminococcaceae;g_*Oscillospira*	0.00012	0.003	0.152
ICAM-1	p_Firmicutes;c_Erysipelotrichi;o_Erysipelotrichales;f_Erysipelotrichaceae	0.00060	<0.001	<0.001
ICAM-1	p_Firmicutes;c_Clostridia;o_Clostridiales;f_Clostridiaceae;g_*SMB53*	-0.00007	<0.001	0.003
ICAM-1	p_Firmicutes;c_Clostridia;o_Clostridiales;f_Peptostreptococcaceae	-0.00040	<0.001	0.013
ICAM-1	p_Firmicutes;c_Clostridia;o_Clostridiales;f_Ruminococcaceae;g_*Ruminococcus*	0.00040	<0.001	0.020
ICAM-1	p_Proteobacteria;c_Alphaproteobacteria;o_Rhizobiales;f_Bradyrhizobiaceae;g_*Bradyrhizobium*	-0.00004	0.003	0.156

A positive coefficient means gut microbial abundance increases with respective variable, while a negative coefficient means gut microbial abundance decreases with respective variable. For readability the kingdom label is not present. "ICAM-1", intercellular adhesion molecule-1; "VCAM-1", vascular cell adhesion molecule. The name of a higher taxon level was added before its taxon abbreviation. "p", phylum; "c", class; "o", order; "f". family; "g", genus.

Table S7. Taxonomic composition of gut microbiota with different metadata in obese adolescents.

Variable	Feature	Coefficient	*p*-value	q-value
Cholesterol	p_Firmicutes;c_Clostridia;o_Clostridiales;f_Lachnospiraceae;g_*Ruminococcus*	0.00114	0.0040	0.192
HDL	p_Firmicutes;c_Clostridia;o_Clostridiales;f_Mogibacteriaceae	0.00103	0.0060	0.239
LDL	p_Firmicutes;c_Clostridia;o_Clostridiales;f_Tissierellaceae;g_*Parvimonas*	-0.00033	0.0012	0.146
ICAM-1	p_Bacteroidetes;c_Bacteroidia;o_Bacteroidales;f__Bacteroidaceae;g_*Bacteroides*	0.00110	0.0001	0.102

A positive coefficient means gut microbial abundance increases with respective variable, while a negative coefficient means gut microbial abundance decreases with respective variable. For readability the kingdom label is not present. "HDL", high-density lipoprotein," LDL", low-density protein, "ICAM-1", intercellular adhesion molecule-1. The name of a higher taxon level was added before its taxon abbreviation. "p", phylum; "c", class; "o", order; "f". family; "g", genus.

Table S8. List of bacterial taxa with their OTUs ID in normal weight children for Figure S1.

**No.**	OTUs ID	Bacteria	No.	OTUs ID	Bacteria
1	10496	Lachnospiraceae	46	370154	Ruminococcaceae
2	12246	*Bacteroides*	47	409732	*Blautia*
3	12426	*Bilophila*	48	508984	Lachnospiraceae
4	14479	Ruminococcaceae	49	519765	Lachnospiraceae
5	14550	Erysipelotrichaceae	50	523782	*Coprococcus*
6	15355	*Blautia*	51	529740	*Roseburia*
7	22335	Rikenellaceae	52	529940	Ruminococcaceae
8	179384	Lachnospiraceae	53	530094	Ruminococcaceae
9	182107	Lachnospiraceae	54	531582	*Blautia*
10	188887	*Bacteroides*	55	532771	Lachnospiraceae
11	190358	Ruminococcaceae	56	535549	*Bacteroides*
12	190975	Barnesiellaceae	57	548233	*Clostridiales*
13	192406	Ruminococcaceae	58	548503	Lachnospiraceae
14	193041	*Blautia*	59	552380	*Ruminococcus*
15	194341	*Bacteroides*	60	556835	*Phascolarctobacterium*
16	194395	Barnesiellaceae	61	560535	Ruminococcaceae
17	194909	*Bacteroides*	62	570507	*Blautia*
18	195222	Ruminococcaceae	63	577170	*Bacteroides*
19	198145	*Blautia*	64	579561	*Blautia*
20	208739	*Faecalibacterium*	65	580008	Erysipelotrichaceae
21	210371	Ruminococcaceae	66	580629	*Bacteroides*
22	264552	*Dialister*	67	581003	*Clostridiales*
23	267718	*Faecalibacterium*	68	583089	*Blautia*
24	287951	*Clostridiales*	69	591891	Lachnospiraceae
25	297150	Lachnospiraceae	70	593422	*Blautia*
26	301910	Lachnospiraceae	71	659361	*Roseburia*
27	316761	*Bacteroides*	72	696563	*Blautia*
28	318970	*Blautia*	73	700298	*Dorea*
29	322380	Ruminococcaceae	74	702414	Lachnospiraceae
30	325244	Lachnospiraceae	75	708680	*Clostridiales*
31	337167	Ruminococcaceae	76	712677	Peptostreptococcaceae
32	347115	*Blautia*	77	727140	*Clostridiales*
33	351231	*Bacteroides*	78	804526	*Blautia*
34	355837	Ruminococcaceae	79	851865	Ruminococcaceae
35	358410	Ruminococcaceae	80	875709	Lachnospiraceae
36	359359	Ruminococcaceae	81	1105552	Lachnospiraceae
37	360158	Lachnospiraceae	82	1106861	*Coprococcus*
38	361966	*Faecalibacterium*	83	1107327	Lachnospiraceae
39	362968	Ruminococcaceae	84	1111458	Peptostreptococcaceae
40	365496	*Bacteroides*	85	2198356	*Bacteroides*
41	365717	*Faecalibacterium*	86	2388088	*Phascolarctobacterium*
42	368117	Ruminococcaceae	87	3678349	*Streptococcus*
43	369027	Lachnospiraceae	88	4338624	*Sutterella*
44	369109	Ruminococcaceae	89	4476780	Rikenellaceae
45	369555	*Ruminococcus*			

Table S9. List of bacterial taxa with their OTUs ID in normal weight adolescents for Figure S2.

No.	OTUs ID	Bacteria	No.	OTUs ID	Bacteria
1	583	*Dialister*	25	521982	Rikenellaceae
2	1060	*Prevotella*	26	523782	*Coprococcus*
3	8103	Ruminococcaceae	27	527751	Lachnospiraceae
4	9799	Bifidobacteriaceae	28	529940	Ruminococcaceae
5	10094	Lachnospiraceae	29	530094	Ruminococcaceae
6	10254	Ruminococcaceae	30	531582	*Blautia*
7	10888	*Ruminococcus*	31	548503	Lachnospiraceae
8	178664	*Clostridiales*	32	552380	*Ruminococcus*
9	180082	*Parabacteroides*	33	560535	Ruminococcaceae
10	182431	Ruminococcaceae	34	570507	Lachnospiraceae
11	190358	Ruminococcaceae	35	577170	*Bacteroides*
12	192365	*Coprococcus*	36	579561	*Blautia*
13	195222	Ruminococcaceae	37	580629	*Bacteroides*
14	198866	*Parabacteroides*	38	583089	*Blautia*
15	228199	*Ruminococcus*	39	591891	Lachnospiraceae
16	264552	*Dialister*	40	593422	*Blautia*
17	272587	*Dialister*	41	700298	Lachnospiraceae
18	287951	*Clostridiales*	42	702414	Lachnospiraceae
19	325244	Lachnospiraceae	43	708680	*Clostridiales*
20	368117	Ruminococcaceae	44	712677	*Clostridiales*
21	369027	Lachnospiraceae	45	851865	Ruminococcaceae
22	369555	*Ruminococcus*	46	1105552	Lachnospiraceae
23	370086	Ruminococcaceae	47	4314092	*Prevotella*
24	370154	Ruminococcaceae			

Table S10. List of bacterial taxa with their OTUs ID in obese children for Figure S3.

**No.**	OTUs ID	Bacteria	No.	OTUs ID	Bacteria
1	335	*Phascolarctobacterium*	46	362214	*Bifidobacterium*
2	1899	*Peptococcus*	47	362968	Ruminococcaceae
3	4007	*Peptococcus*	48	365496	*Bacteroides*
4	6529	*Lactobacillus*	49	365717	*Faecalibacterium*
5	7389	*Haemophilus*	50	369027	Lachnospiraceae
6	8433	Clostridiales	51	370086	Ruminococcaceae
7	9453	*Prevotella*	52	370154	Ruminococcaceae
8	11321	*Phascolarctobacterium*	53	403701	*Dialister*
9	11423	*Bacteroides*	54	517282	*Blautia*
10	12345	*Phascolarctobacterium*	55	521982	Clostridiales
11	13188	*Prevotella*	56	522364	*Prevotella*
12	13949	Ruminococcaceae	57	523782	*Coprococcus*
13	14550	Erysipelotrichaceae	58	524371	*Prevotella*
14	19296	*Bacteroides*	59	524884	*Eubacterium*
15	19314	*Parabacteroides*	60	529940	Ruminococcaceae
16	21736	*Phascolarctobacterium*	61	530094	Ruminococcaceae
17	22231	*Haemophilus*	62	531582	*Blautia*
18	22619	*Peptococcus*	63	531928	Clostridiales
19	22894	Ruminococcaceae	64	560535	Ruminococcaceae
20	24722	*Bacteroides*	65	567846	Bifidobacterium
21	41229	*Sutterella*	66	570507	*Blautia*
22	130864	*Lactobacillus*	67	574111	*Prevotella*
23	157424	*Phascolarctobacterium*	68	580008	Erysipelotrichaceae
24	179261	*Sutterella*	69	583746	*Dialister*
25	180082	*Parabacteroides*	70	587753	Coriobacteriaceae
26	183480	Rikenellaceae	71	591891	Lachnospiraceae
27	183603	*Bacteroides*	72	593422	*Blautia*
28	190358	Ruminococcaceae	73	639310	*Bifidobacterium*
29	190975	Barnesiellaceae	74	700298	*Dorea*
30	193723	Bacteroidales	75	702414	Lachnospiraceae
31	194395	Barnesiellaceae	76	708680	Clostridiales
32	195222	Ruminococcaceae	77	712677	Peptostreptococcaceae
33	196604	*Catenibacterium*	78	716286	*Lactobacillus*
34	198866	*Parabacteroides*	79	804526	*Blautia*
35	202162	Ruminococcaceae	80	849535	*Prevotella*
36	208739	Faecalibacterium	81	850218	Phascolarctobacterium
37	215331	*Peptococcus*	82	851865	Ruminococcaceae
38	230403	*Bacteroides*	83	875709	Lachnospiraceae
39	235591	*Lactobacillus*	84	1105328	*Blautia*
40	236308	*Lactobacillus*	85	1105552	Lachnospiraceae
41	269937	*Prevotella*	86	1106861	*Coprococcus*
42	293883	*Phascolarctobacterium*	87	1107327	Lachnospiraceae
43	309133	Enterococcaceae	88	1111458	Peptostreptococcaceae
44	339685	*Peptococcus*	89	4226929	*Bacteroides*
45	361966	*Faecalibacterium*			

Table S11. List of bacterial taxa with their OTUs ID in obese adolescents for Figure S4.

**No.**	OTUs ID	Bacteria	No.	OTUs ID	Bacteria
1	1002	*Sphingomonas*	51	362214	*Bifidobacterium*
2	1184	*Collinsella*	52	362968	Ruminococcaceae
3	4568	*Bradyrhizobium*	53	363794	*Collinsella*
4	5337	Clostridiaceae	54	365496	*Bacteroides*
5	6613	Bradyrhizobium	55	365717	*Faecalibacterium*
6	7367	*Prevotella*	56	368175	*Collinsella*
7	7786	Clostridiales	57	369109	Ruminococcaceae
8	9022	*Lactobacillus*	58	370154	Ruminococcaceae
9	9328	*Collinsella*	59	383885	*Lactobacillus*
10	10094	Lachnospiraceae	60	469663	*Atopobium*
11	10254	Ruminococcaceae	61	470382	Clostridiales
12	10278	*Lactobacillus*	62	471180	Bifidobacterium
13	10560	*Collinsella*	63	529940	Ruminococcaceae
14	11912	Clostridiaceae	64	530094	Ruminococcaceae
15	12479	*Lactobacillus*	65	531582	*Blautia*
16	12723	Lachnospiraceae	66	556835	*Phascolarctobacterium*
17	12734	Ruminococcaceae	67	560336	*Bacteroides*
18	12974	*Lactobacillus*	68	560535	Ruminococcaceae
19	13485	*Bifidobacterium*	69	566154	Coriobacteriaceae
20	29566	*Sneathia*	70	567846	*Bifidobacterium*
21	128300	*Lactobacillus*	71	568118	*Prevotella*
22	130468	*Lactobacillus*	72	570507	*Blautia*
23	130864	*Lactobacillus*	73	579608	*Streptococcus*
24	133372	*Parvimonas*	74	583089	*Blautia*
25	137183	Bifidobacteriaceae	75	584347	*Actinomyces*
26	177679	S24-7	76	589071	*Bacteroides*
27	183988	Clostridiales	77	593422	*Blautia*
28	188956	*Blautia*	78	663885	*Prevotella*
29	190358	Ruminococcaceae	79	696563	*Blautia*
30	191112	Lachnospiraceae	80	708680	Clostridiales
31	195222	Ruminococcaceae	81	712047	Clostridiaceae
32	197060	Clostridiaceae	82	712677	Clostridiales
33	198915	*Blautia*	83	826270	*Bradyrhizobium*
34	208739	*Faecalibacterium*	84	826382	Lachnospiraceae
35	225846	*Dialister*	85	840914	*Prevotella*
36	236308	*Lactobacillus*	86	844589	S24-7
37	272516	*Adlercreutzia*	87	851726	*Megasphaera*
38	292921	*Prevotella*	88	851865	Ruminococcaceae
39	296420	Clostridiaceae	89	858535	Coriobacteriaceae
40	318990	Lachnospiraceae	90	866365	Caulobacteraceae
41	322367	Clostridiaceae	91	934235	Rhizobiales
42	338273	Clostridiaceae	92	986513	*Clostridium*
43	338381	*Prevotella*	93	1105328	*Blautia*
44	344114	Clostridiales	94	1105552	Lachnospiraceae
45	354905	*Lactobacillus*	95	1106861	*Coprococcus*
46	357046	Rikenellaceae	96	1109964	Sphingomonadaceae
47	358798	Lachnospiraceae	97	1111458	Peptostreptococcaceae
48	359359	Ruminococcaceae	98	1656781	*Prevotella*
49	360158	Lachnospiraceae	99	64179	*Sphingomonas*
50	360518	*Bacteroides*			

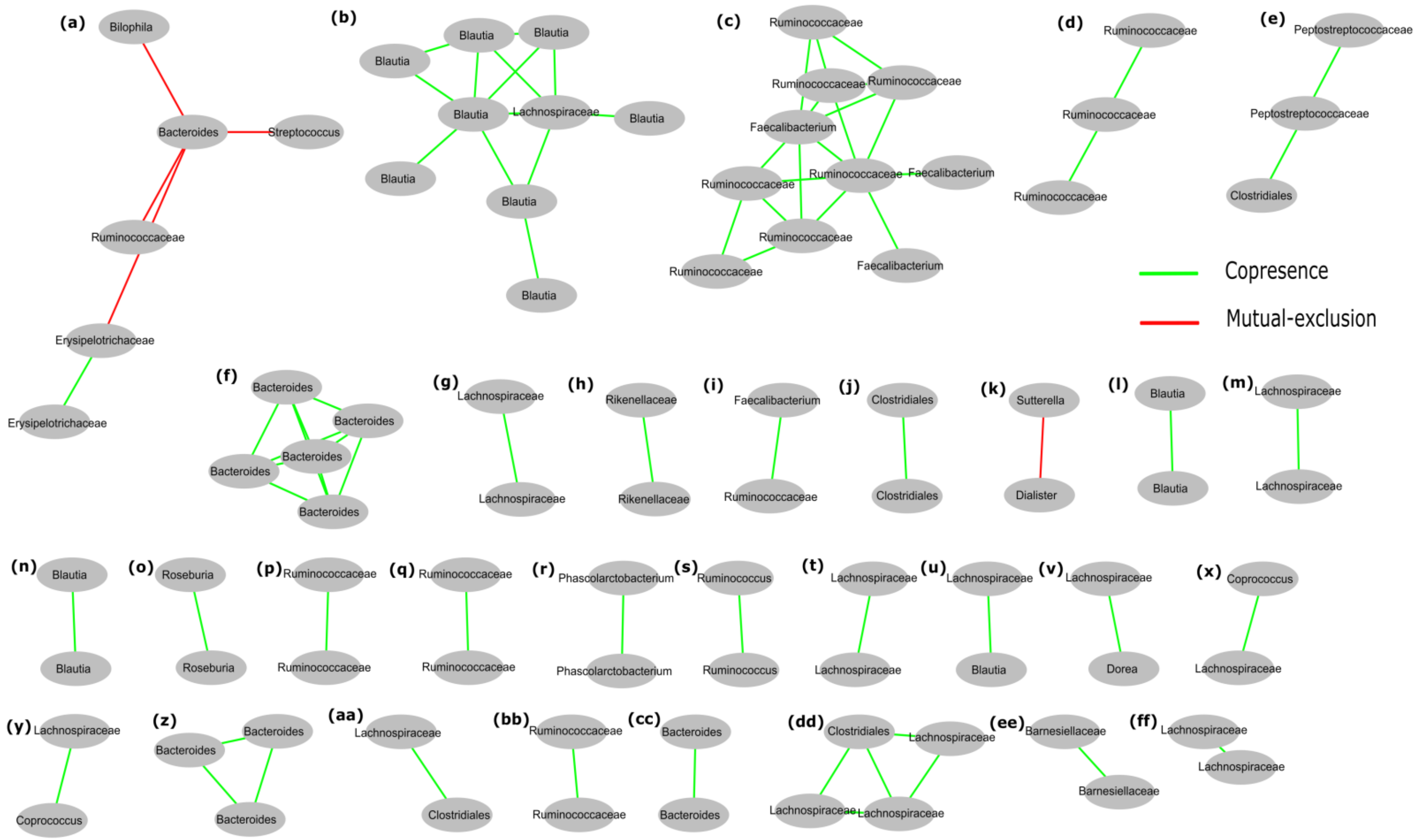

Figure S1. Significant co-occurrence analysis between gut microbiota in normal weight Mexican children (**a-ff**). This graphic shows the interesting interaction between different bacterial communities; copresence (positive interaction; *green lines*). This analysis was performed using *otu_table.biom* file in CoNet plugin tool and generated co-occurrence networks were visualized by Cytoscape (v3.6.1) software as described in Material and methods. Each node indicates a microbial clade (bacterial taxon) belonging to a unique OTUs number. Edges (lines) are connecting two nodes, represent significant correlations ($p < .05$; $q < 0.05$; $R > 0.8$). In this graphics some bacteria names are shown more than one time e.g. Ruminococcaceae etc., however they belong to different OTUs number. Bacteria names and corresponding OTUs number are shown in Table S8, and the significant interaction values are shown in Table S12.

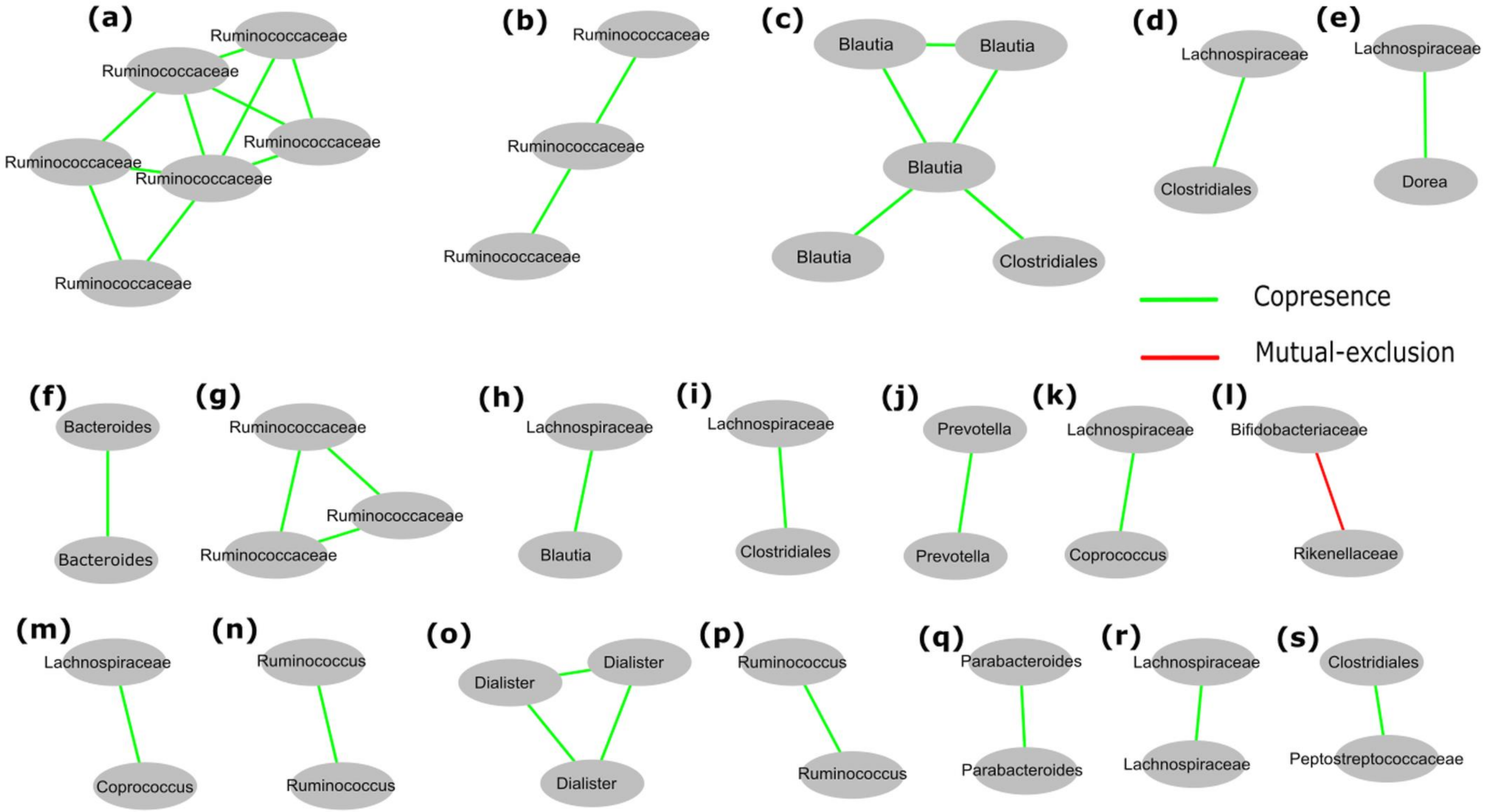

Figure S2. Significant co-occurrence analysis between gut microbiota in normal weight Mexican adolescents (**a-s**). This graphic shows the interesting interaction between different bacterial communities; copresence (positive interaction; *green lines*). This analysis was performed using *otu_table.biom* file in CoNet plugin tool and generated co-occurrence networks were visualized by Cytoscape (v3.6.1) software as described in Material and methods. Each node indicates a microbial clade (bacterial taxon) belonging to a unique OTUs number. Edges (lines) are connecting two nodes, represent significant correlations ($p < .05$; $q < 0.05$; $R > 0.8$). In this graphics some bacteria names are shown more than one time e.g. Ruminococcaceae etc., however they belong to different OTUs number. Bacteria names and corresponding OTUs number are shown in Table S9, and the significant interaction values are shown in Table S12.

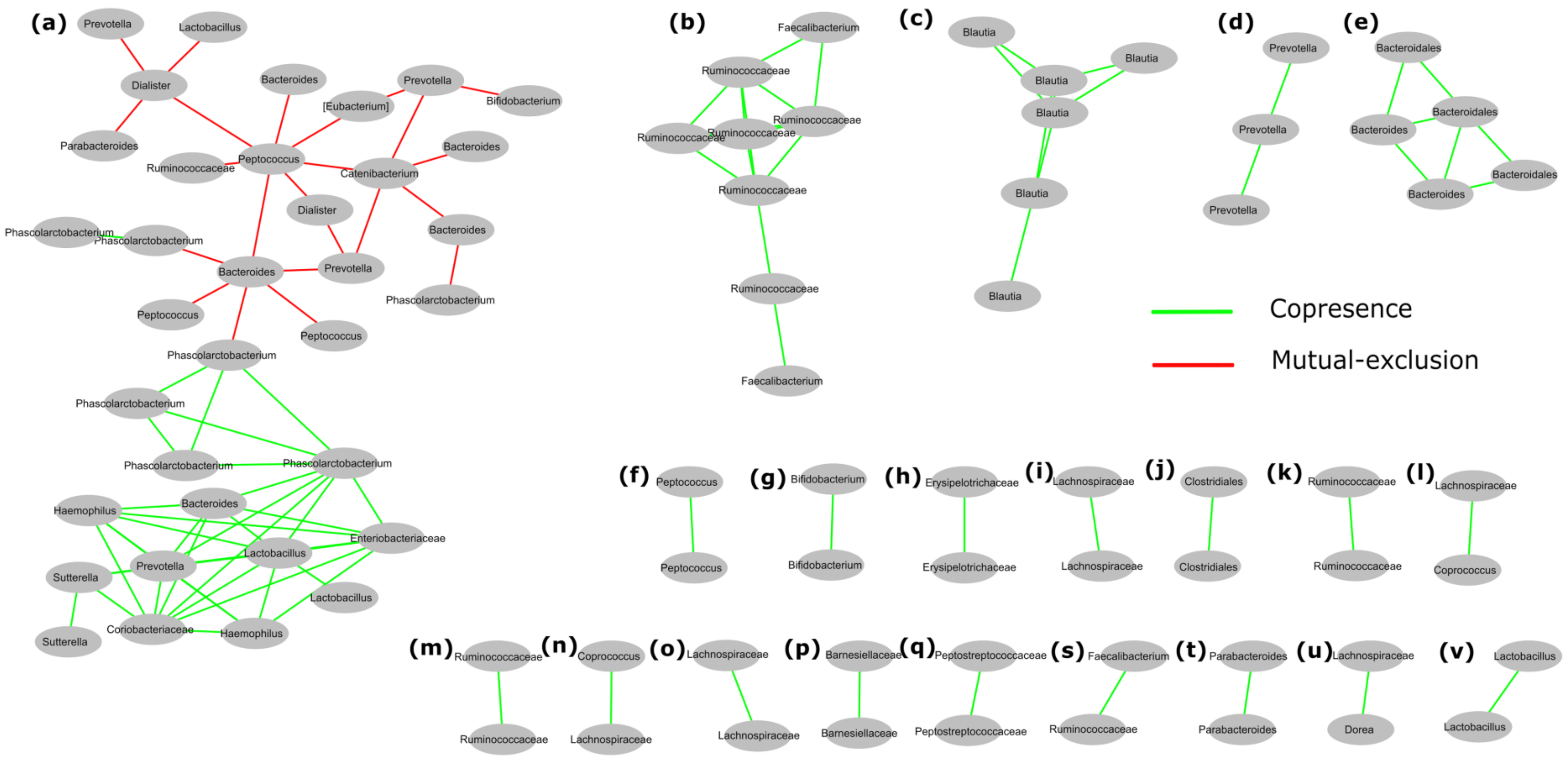

Figure S3. Significant co-occurrence analysis between gut microbiota in obese Mexican children (**a-v**). This graphic shows the interesting interaction between different bacterial communities; copresence (positive; *green lines*). This analysis was performed using *otu_table.biom* file in CoNet plugin tool and generated co-occurrence networks were visualized by Cytoscape (v3.6.1) software as described in Material and methods. Each node indicates a microbial clade (bacterial taxon) belonging to a unique OTUs number. Edges (lines) are connecting two nodes, represent significant correlations ($p < .05$; $q < 0.05$; $R > 0.8$). In this graphics some bacteria names are shown more than one time e.g. Ruminococcaceae etc., however they belong to different OTUs number. Bacteria names and corresponding OTUs number are shown in Table S10, and the significant interaction values are shown in Table S12.

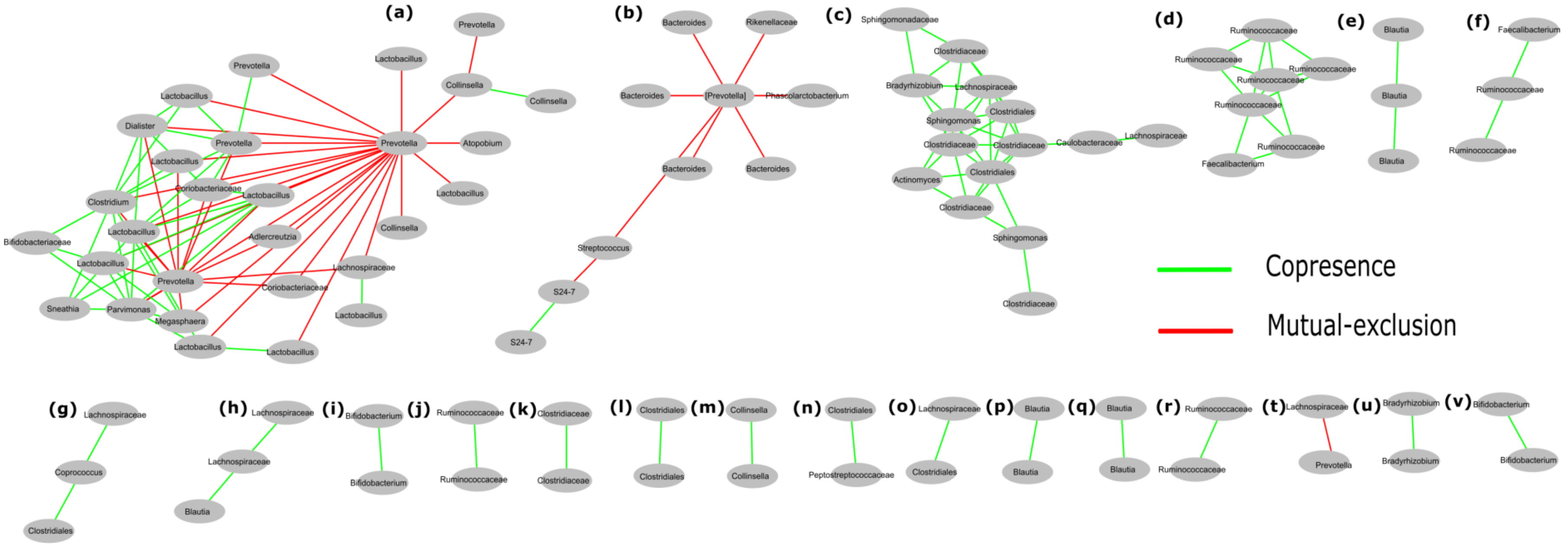

Figure S4. Significant co-occurrence analysis between gut microbiota in obese Mexican adolescents (**a-v**). This graphic shows the interesting interaction between different bacterial communities; copresence (positive interaction; *green lines*). This analysis was performed using *otu_table.biom* file in CoNet plugin tool and generated co-occurrence networks were visualized by Cytoscape (v3.6.1) software as described in Material and methods. Each node indicates a microbial clade (bacterial taxon) belonging to a unique OTUs number. Edges (lines) are connecting two nodes, represent significant correlations ($p < .05$; $q < 0.05$; $R > 0.8$). In this graphics some bacteria names are shown more than one time e.g. Ruminococcaceae etc., however they belong to different OTUs number. Bacteria names and corresponding OTUs number are shown in Table S11, and the significant interaction values are shown in Table S12.

-----end-of-text-----
